# Supplementary material for: PD-L1- and IL-4-expressing basophils promote pathogenic accumulation of T follicular helper cells in lupus
Source: Nat Commun. 2024 Apr 22;15:3389. doi: 10.1038/s41467-024-47691-w (PMC11035650; doi:10.1038/s41467-024-47691-w)
Supplement: Supplementary file 1 — Supplementary Information [file 41467_2024_47691_MOESM1_ESM.pdf]

PD-L1- and IL-4-expressing basophils promote pathogenic accumulation of T follicular helper cells in lupus.

SUPPLEMENTARY INFORMATION

Authors: John TCHEN [1,2*], Quentin SIMON [1,2,3*], Léa CHAPART [1,2], Morgane K. THAMINY [1,2], Shamila VIBHUSHAN [1,2], Loredana SAVEANU [1,2], Yasmine LAMRI [1,2], Fanny SAIDOUNE [1,2], Emeline PACREAU [1,2], Christophe PELLEFIGUES [1,2], Julie BEX-COUDRAT [1,2], Hajime KARASUYAMA [4], Kensuke MIYAKE [4], Juan HIDALGO [5], Padraic G. FALLON [6], Thomas PAPO [1,2,7], Ulrich BLANK [1,2], Marc BENHAMOU [1,2], Guillaume HANOUNA [1,2,8], Karim SACRE [1,2,7], Eric DAUGAS [1,2,8] and Nicolas CHARLES [1,2†]

[1] Université Paris Cité, Centre de Recherche sur l'Inflammation, INSERM UMR1149, CNRS EMR8252, Faculté de Médecine site Bichat,75018, Paris, France

[2] Université Paris Cité, Laboratoire d'Excellence Inflamex, 75018, Paris, France

[3] Inovarion, 75005, Paris, France

[4] Inflammation, Infection and Immunity Laboratory, TMDU Advanced Research Institute, Tokyo Medical and Dental University (TMDU), Tokyo, Japan

[5] Universidad Autonoma de Barcelona, Facultad de Biociencias, Unidad de Fisiologia Animal Bellaterra, Bellaterra Campus 08193, Barcelona, Spain

[6] School of Medicine, Trinity College Dublin, Dublin 2, Ireland.

[7] Service de Médecine Interne, Hôpital Bichat, Assistance Publique – Hôpitaux de Paris, 75018, Paris, France

[8] Service de Néphrologie, Hôpital Bichat, Assistance Publique – Hôpitaux de Paris, 75018, Paris, France

* Co-first authors

†**Corresponding author**:

Nicolas Charles, PhD

Centre de Recherche sur l'Inflammation, INSERM UMR1149, CNRS EMR8252,

Université Paris Cité, Faculté de Médecine site Bichat,

16 rue Henri Huchard, 75018 Paris, France.

Phone: +33 157277306

E-mail: nicolas.charles@inserm.fr

ORCID : **0000-0002-5416-5834**

Supplementary Figure 1

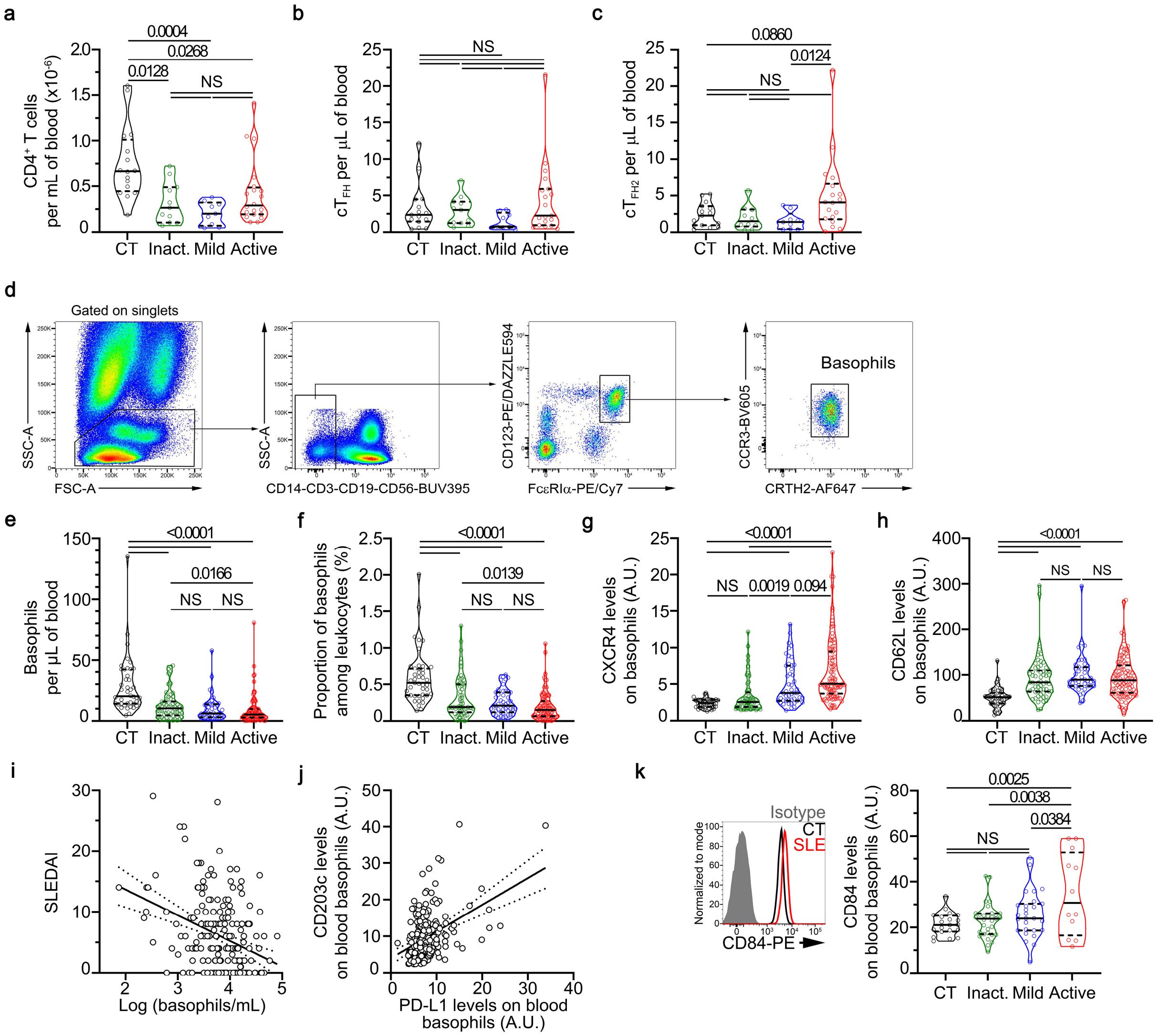

Supplementary Fig. 1: Activated phenotype of basophils in SLE patients

(**a**-**c**) Absolute number of CD4+ T cells (**a**), cTFH cells (**b**) and cTFH2 cells (**c**) per volume of blood in samples from healthy controls (CT) and inactive (inact.), mild or active SLE patients (n= 16/10/12/20, respectively) was determined by flow cytometry as described in **Fig. 1a**. (**d**) Flow cytometry gating strategy used to define basophils in human blood samples. Basophils were defined as $SSC^{lo}CD14^{-}CD3^{-}CD19^{-}CD56^{-}CD123^{+}Fc\varepsilon RI\alpha^{+}CCR3^{+}CRTH2^{+}$ cells. (**e**) Absolute number of basophils per µL of blood in samples from healthy controls (CT) and inactive (inact.), mild or active SLE patients (n= 42/61/47/96, respectively) was determined by flow cytometry as described in (**d**). (**f**) Proportions (%) of basophils among leukocytes in blood samples as described in (**d**,**e**) (n= 43/61/47/98). (**g**) CXCR4 expression levels on blood basophils as described in (**d**,**e**) (n= 43/61/47/98, respectively) as determined by flow cytometry. (**h**) CD62L expression levels on blood basophils as described in (**d**,**e**) (n= 43/59/44/98, respectively) as determined by flow cytometry. (**i**) Correlation (and linear regression with 95% confidence intervals) between Log (basophil numbers per mL of blood) and SLEDAI (Spearman r = - 0.3320, $p < 0.0001$, n= 201). (**j**) Correlation (and linear regression with 95% confidence intervals) between basophil CD203c and

PD-L1 expression levels (Spearman r=0.3113, $p < 0.0001$, n= 204). (**k**) **Left**, Representative FACS analysis of CD84 expression levels on blood basophils from a healthy control (CT, black line), a patient with active SLE (red line), and isotype control staining (grey filled histogram). **Right**, CD84 expression levels on blood basophils from healthy controls (CT) and inactive (inact.), mild or active SLE patients (n= 20/31/27/12, respectively) as determined by flow cytometry. (**a**-**c**, **e**-**h**, **k**) Data are presented as violin plots with median (solid line) and quartiles (dotted lines). Statistical analyses were Kruskal-Wallis tests followed by Dunn's multiple comparisons tests. *P* values are shown above each bracket. NS: not significant. A.U. arbitrary units. Source data are provided in the Source Data file.

Supplementary Figure 2

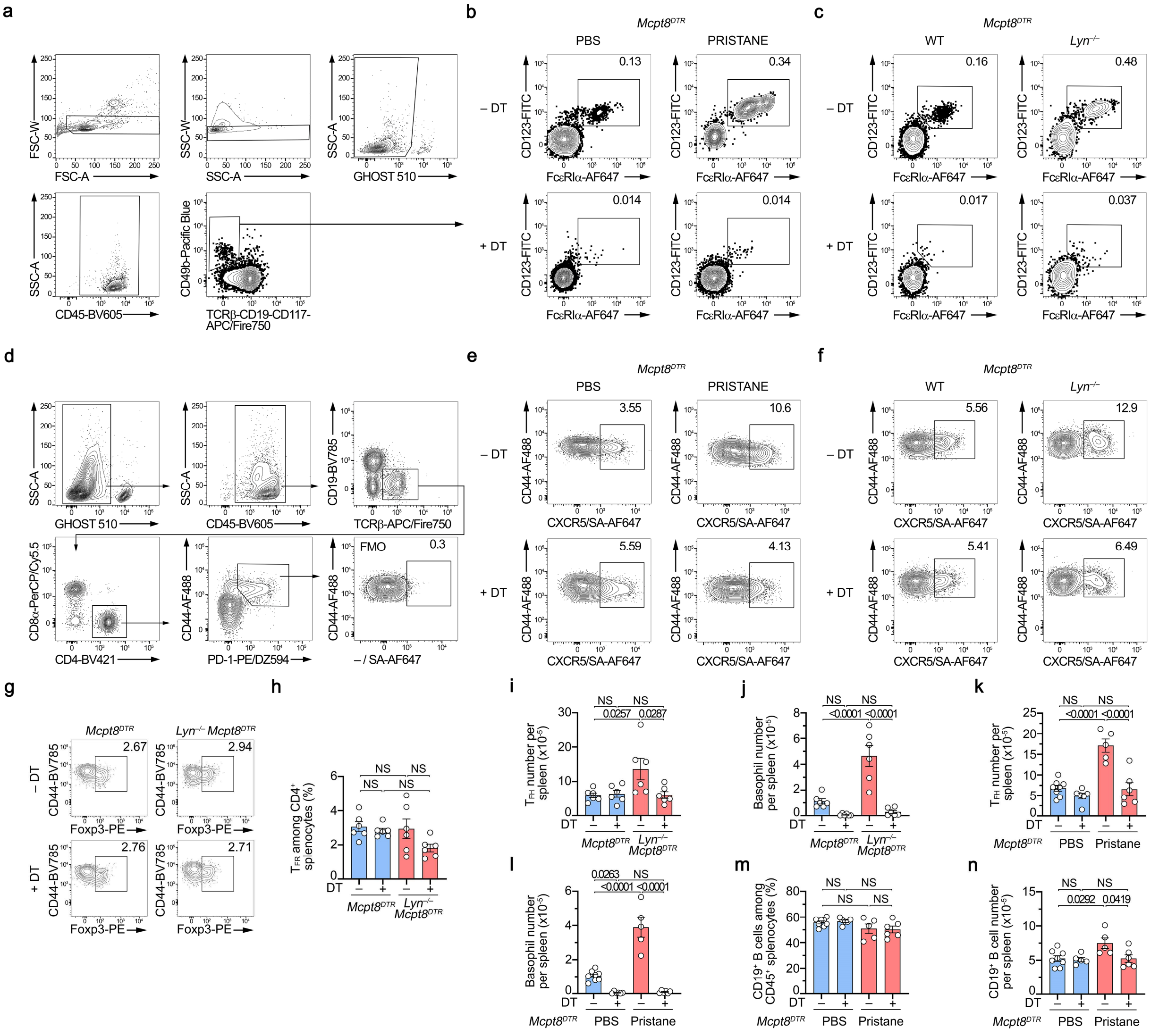

Supplementary Fig. 2 : Basophil and TFH gating strategy and effects of DT injection in lupus-like mouse models on *Mcpt8*^{DTR} background

(**a**) Contour plots showing the gating strategy used to define basophils prior to the final step shown in (b,c). (**b**,**c**) Representative contour plots showing gating and proportions of basophils (defined as CD45^{+} TCRβ^{-} CD19^{-} CD117^{-} CD49b^{+} CD123^{+} FcεRIα^{+} cells) among CD45^{+} splenocytes in *Mcpt8*^{DTR} mice PBS- or pristane-injected (**b**) and in aged *Mcpt8*^{DTR} (WT) and *Lyn*$^{-/-}$ *Mcpt8*^{DTR} (*Lyn*$^{-/-}$) mice (**c**) basophil-depleted (+DT) or not (–DT). Gating strategy used in **Fig. 2a,b,e,f** and **Fig. 3q-u**. (**d-f**) Contour plots showing the gating strategy and proportions of TFH cells (defined as CD45^{+} CD19^{-} TCRβ^{+} CD8α^{-} CD4^{+} CD44^{+} PD-1^{+} CXCR5^{+} cells) among spleen CD4^{+} T cells (**e**,**f**) from mice as described in (**b**,**c**). CXCR5 positivity was determined on the fluorescence minus one (FMO, **d**). Gating strategy used in **Fig. 2c**,**d**,**l**; **Fig. 3a-f**,**k-p**; **Fig. 4a-i**; **Fig. 6a**; **Fig. 7a** and **Fig. 8g** as well as in **Supplementary Fig. 3c**,**f**; **4 b**,**c**; **6a-d**; **7a**,**e-l**; **8f**; **9a**,**b** and **11e**. (**g**) Representative contour plots showing gating and proportions of regulatory TFH cells (TFR) (defined as Foxp3+ TFH cells) among spleen CD4+ T cells from mice as described in **Fig. 2d**. (**h**) Proportions (%) of TFR among CD4^{+} splenocytes in mice as in (**f**) (n= 6 per group). (**i**,**j**) Absolute numbers of TFH (**i**) and basophils (**j**) per spleen from mice as in (**c**) (n= 6 per group). (**j**,**k**) Absolute numbers of TFH (**k**) and basophils (**l**) per

spleen from mice as in (**b**) (n= 8/6/5/6). (m,**n**) Proportions among CD45$^+$ splenocytes (%) (**m**) (n= 8/6/5/6) and absolute numbers per spleen (**n**) (n= 8/5/5/6) of CD19$^+$ B cells from mice as in (**b**). Results are from at least three independent experiments and presented as individual values in bars representing the mean values ± s.e.m. Statistical analyses were done by two-way ANOVA test followed by Tukey's multiple comparisons test between the indicated groups. *P* values are shown above each bracket. NS: not significant. SA: streptavidin. Source data are provided in the Source Data file.

Supplementary Figure 3

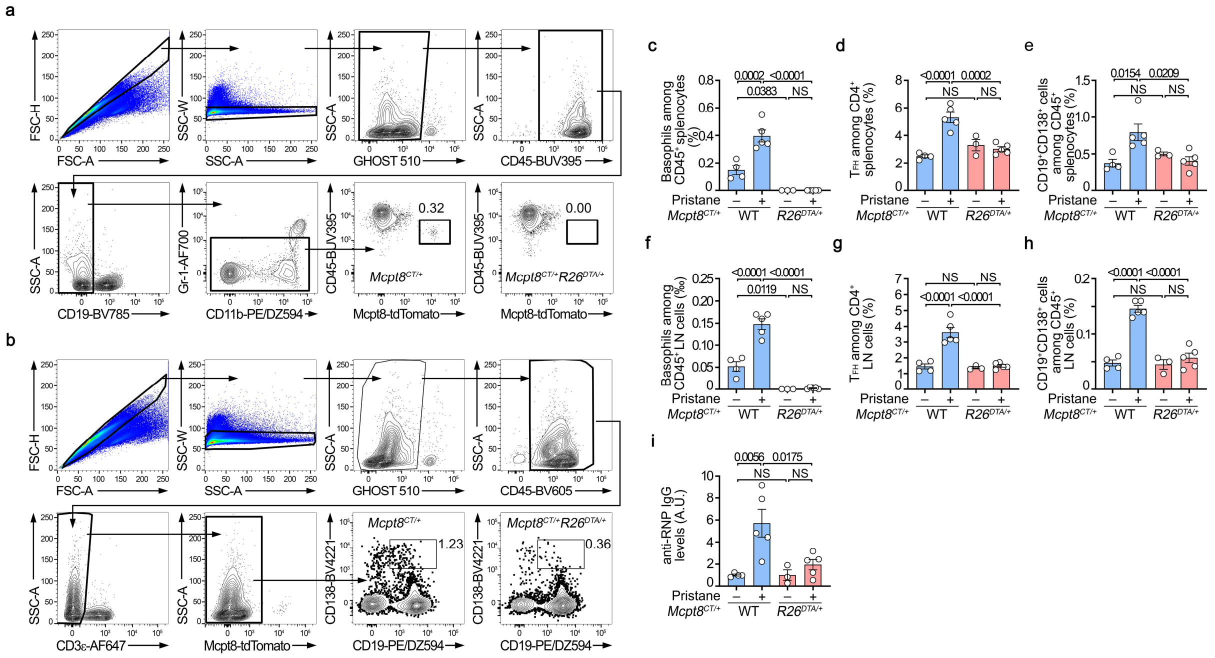

Supplementary Fig. 3: Basophil-TFH functional relationship in lupus-like disease 24 weeks after pristane injection

(**a**) Gating strategy used to define basophils in *Mcpt8*$^{CT/+}$ and all mice bred with them. Gating strategy used in **Fig. 5a**,**b**,**d**-**g**; **Fig. 6b**,**c**; **Fig. 7b**,**d**; **Fig.8a** and as well in **Supplementary Fig. 3**; **7b**-**d**,**k**-**n**; and **8a**,**e**. (**b**) Gating strategy used to define CD19^{+}CD138^{+} cells. Gating strategy used in **Fig. 6e** , **Fig. 7c** and **Supplementary Fig. 3e**,**h**. (**c**,**d**,**e**) Proportions (%) of basophils among CD45^{+} splenocytes (**c**), of TFH among spleen CD4^{+} T cells (**d**), and of CD19^{+}CD138^{+} cells among CD45^{+} splenocytes (**e**) from *Mcpt8*$^{CT/+}$ (WT, blue) and basophil-deficient (*Mcpt8*$^{CT/+}$ *R26*$^{DTA/+}$, red) mice analyzed 24 weeks after PBS or pristane (– or +, respectively) injections. (**f**,**g**,**h**) Proportions (%) of basophils among CD45^{+} lymph node (LN) cells (**f**), of TFH among LN CD4^{+} T cells (**g**), and of CD19^{+}CD138^{+} cells among CD45^{+} LN cells (**h**) from the same mice as in (**c**,**d**,**e**). (**i**) Anti-RNP IgG plasma titers in mice as in (**c**,**d**,**e**) were quantified by ELISA. O.D. values at 450 nm were normalized to the mean of the PBS-injected mice of the same genotype. (**c**-**i**) Results are presented as individual values in bars representing the mean values ± s.e.m. (n= 4/5/3/5). Statistical analyses were done by two-way ANOVA followed by Tukey's multiple comparison tests between the indicated groups. *P* values are shown above each bracket. NS: not significant. A.U.: arbitrary units. Source data are provided in the Source Data file.

Supplementary Figure 4

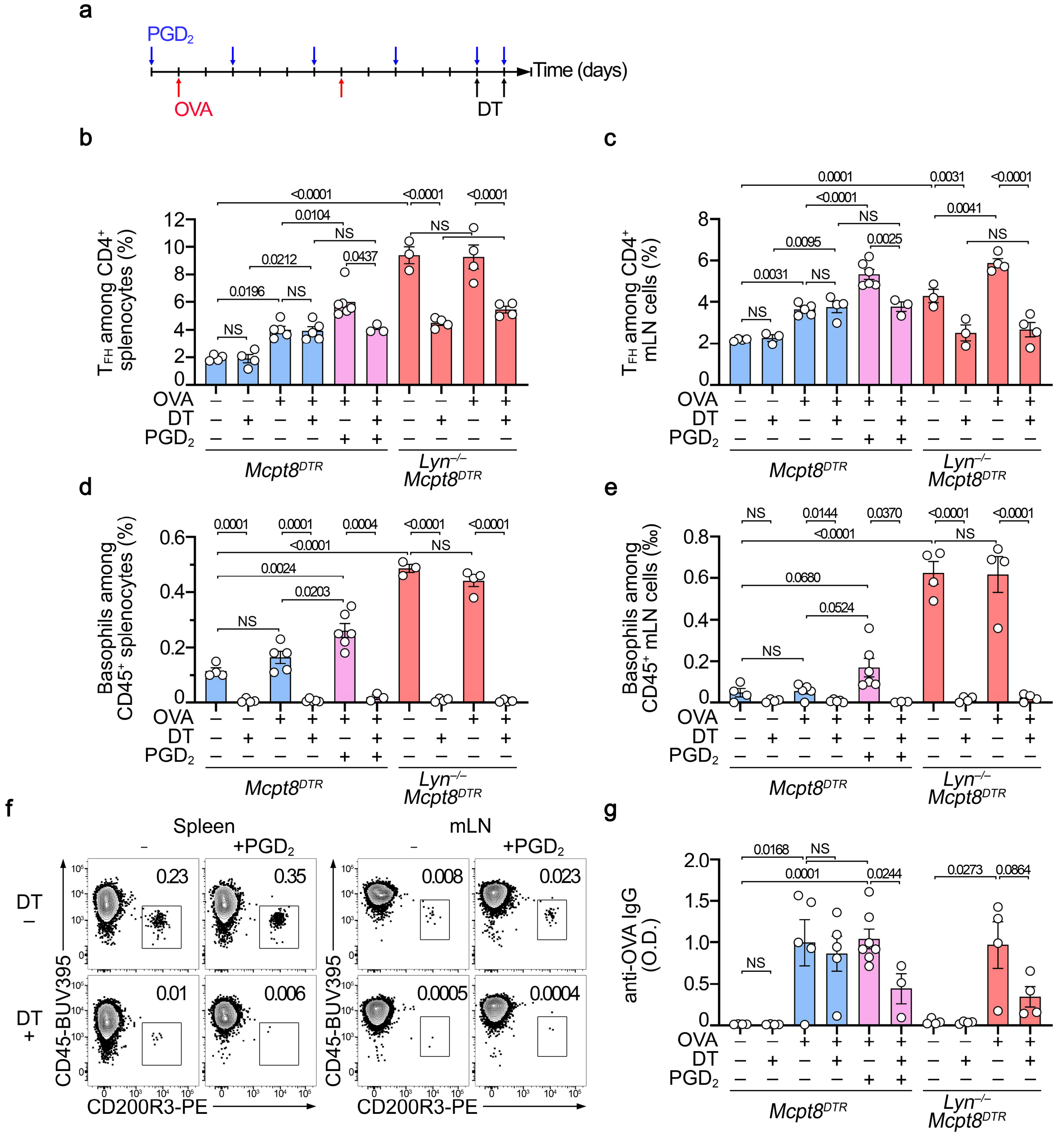

Supplementary Fig. 4: Basophil contribution to ovalbumin immunization in healthy and lupus-like contexts

(**a**) The immunization procedure was by intraperitoneal injection of 200 µL of a 50/50 emulsion of Alum with 100 µg of ovalbumin (OVA) diluted in PBS or with PBS alone for control mice on day 0 and day 7. Concerned mice were intraperitoneally injected with 20 nmoles of PGD_2 in 100 µL of PBS on day -1, 3, 6, 9, 12 and 13. For DT-mediated basophil depletion, 1 µg of DT (or PBS as a control) was injected intraperitoneally in 100 µL of PBS on day 12 and day 13. Analysis was made on day 14. (**b**,**c**) Proportions (%) of TFH cells among $CD4^+$ T cells in spleen (**b**) (n= 4/4/5/5/6/3/3/4/4/4) or mesenteric lymph node (mLN) (**c**) (n= 4/3/5/4/6/3/3/3/4/4) from young (<16 weeks old) *Mcpt8*^{DTR} not treated (blue bars) or

treated with PGD_2 (purple bars, +) or in *Lyn*$^{-/-}$*Mcpt8*^{DTR} (red bars) mice immunized with ovalbumin (OVA +) or not (OVA –) and basophil-depleted (DT +) or not (DT –) 48 hours before analysis. (**d**,**e**) Proportions (% or ‰) of basophils among $CD45^+$ cells in spleen (**d**) (n= 4/4/5/5/6/3/3/4/4/4) or mesenteric lymph node (mLN) (**e**) (n= 4/4/5/5/6/3/4/4/4/4) from the mice described in (**b**,**c**) as measured by flow cytometry as shown in (**f**). (**f**) This gating strategy was used in **Supplementary Fig. 4d**,**e**; **7j**; **11b**,**c**,**d** and in **Fig. 5d** and **8d**,**e**,**f**. (**g**) Anti-OVA IgG plasma levels in mice described in (**b**,**c**) were determined by ELISA. The values presented are 450 nm optical density values (O.D.) (n= 4/4/5/5/7/3/4/4/4/4). (**b**-**g**) Results are from at least three independent experiments and presented as individual values in bars representing the mean values ± s.e.m. Statistical analyses were by one-way ANOVA test followed by Holm-Šídák's multiple comparisons test (**b**,**c**) or two-sided unpaired t tests (**d**,**e,g**) between the indicated groups. *P* values are shown above each bracket. NS: not significant. Source data are provided in the Source Data file.

Supplementary Figure 5

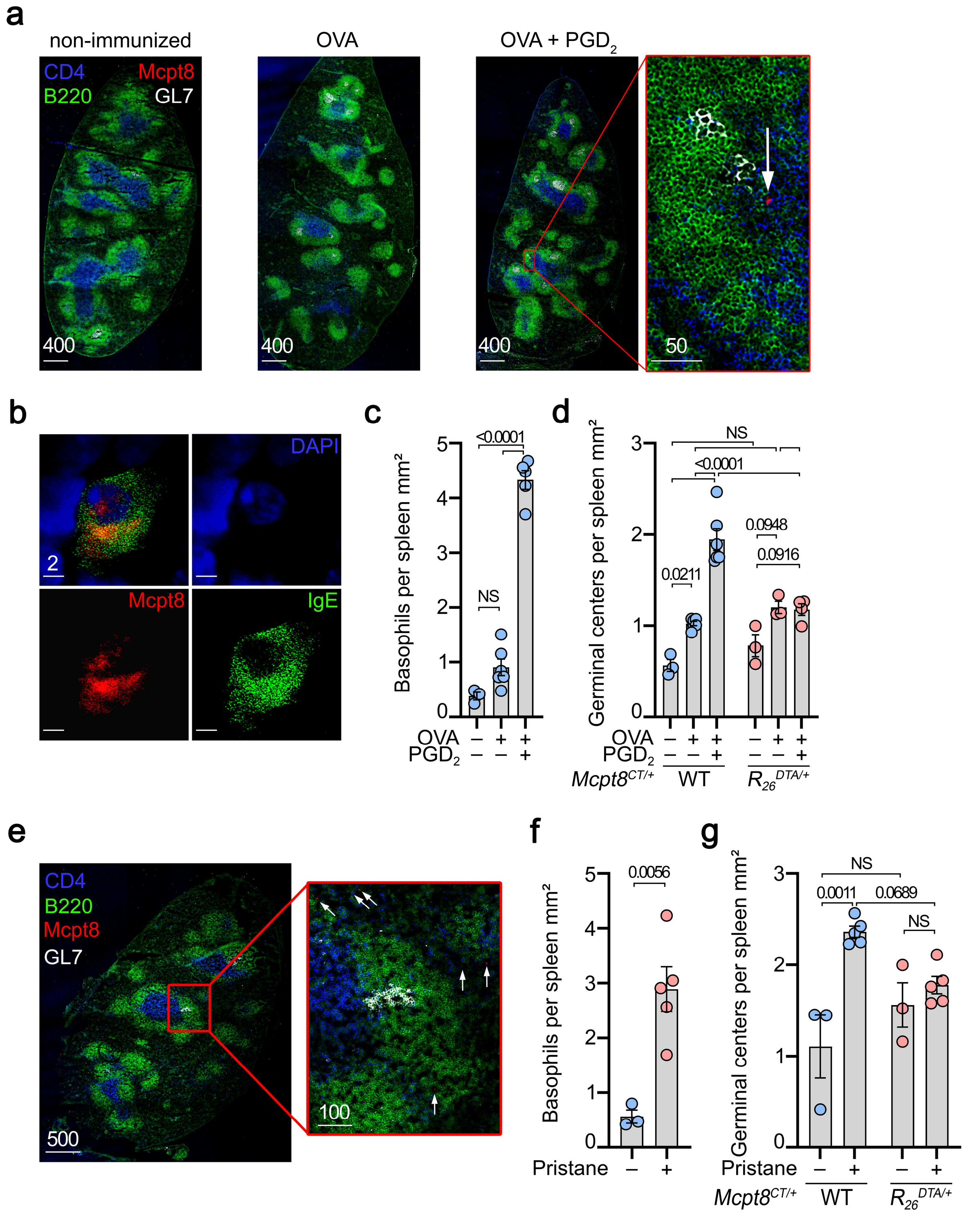

Supplementary Fig. S5: Spleen basophil and germinal center visualization in OVA-immunized mice ± PGD_2 treatment and in pristane-treated mice

(**a**) Immunofluorescent staining of CD4 (blue, CD4$^+$ T cells), B220 (green, B cells), Mcpt8 (red, basophils) and GL7 (white, germinal centers) in spleen sections from *Mcpt8*$^{CT/+}$ mice non-immunized (left),

immunized with OVA (middle) or immunized with OVA and treated with PGD_2 (right) (scale bar = 400 µm), as described in supplementary Fig. S4a. A numerical zoom of the OVA+PGD_2 condition is shown on the right (scale bar = 50 µm) showing a germinal center (GL7, white) and a basophil (red) (indicated by an arrow) at the T:B border. (**b**) Immunofluorescent staining of nuclei (DAPI, blue), IgE (green), and Mcpt8 (red) in a spleen section from a *Mcpt8*$^{CT/+}$ mouse immunized with OVA and treated with PGD_2 showing that Mcpt8 staining is specific for basophils (scale bar = 2 µm). (**c**) Basophil numbers per mm² of spleen sections as depicted in (**a**) were quantified on 1 to 3 sections from at least two mice per group (n =3/6/5). (**d**) Germinal center numbers (defined as GL7$^+$ clusters) per mm² of spleen sections as depicted in (**a**) were quantified on 1 to 3 sections from at least two mice per group from *Mcpt8*$^{CT/+}$ and *Mcpt8*$^{CT/+}$*R*$_{26}$ $^{DTA/+}$ (basophil-deficient) mice treated as described in **Supplementary Fig. 4a** (n =3/6/6/3/3/4). (**e**) Immunoflurescent staining as in (**a**) in a spleen section from a pristane-treated *Mcpt8*$^{CT/+}$ mice (scale bar = 500 µm). A numerical zoom of the indicated area is shown on the right (scale bar = 100 µm). White arrows show Mcpt8$^+$ (red) basophils. (**f**) Basophil numbers per mm² of spleen sections as depicted in (**e**) were quantified on 1 to 3 sections from at least two mice per group (PBS- and pristane-treated mice) (n =3/5). (**g**) Germinal center numbers per mm² of spleen sections as depicted in (**e**) were quantified on 1 to 3 sections from at least two mice per group from *Mcpt8*$^{CT/+}$ and *Mcpt8*$^{CT/+}$*R*$_{26}$ $^{DTA/+}$ (basophil-deficient) mice treated with PBS (–) or pristane (+) (n =3/5/3/5). (**c**,**d**,**f**,**g**) Data are presented as individual values in bars representing the mean values ± s.e.m. (**c**) Statistical analyses were done by one-way (**c**) or two-way (**d**,**g**) ANOVA test followed by Tukey's multiple comparisons test between the indicated groups or by a two-sided unpaired Student's *t* test (**f**). *P* values are shown above each bracket. NS: not significant. Source data are provided in the Source Data file.

Supplementary Figure 6

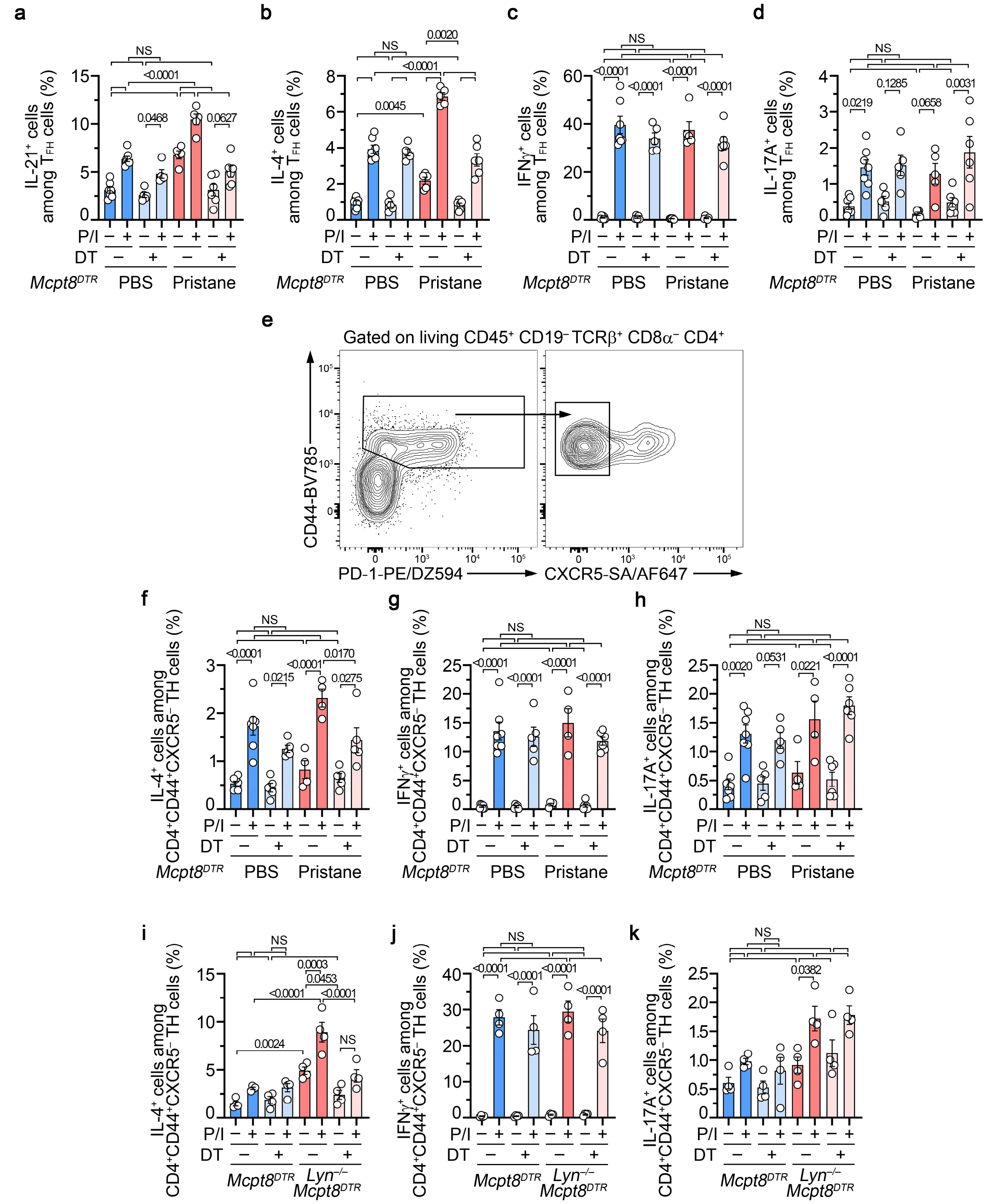

Supplementary Fig. 6: IL-21, IL-4, IFNγ and IL-17 A production by TFH cells and non-TFH CD4+ T Helper cells in basophil-sufficient and basophil-depleted mice in the lupus-like context (related to Fig. 3).

(**a-d**) Proportions (%) of IL-21 (**a**), IL-4 (**b**), IFNγ (**c**) and IL-17A (**d**) producing cells among TFH cells in splenocytes stimulated with with P/I (+) or not (–) from PBS-injected (blue) and pristane-injected *Mcpt8*^{DTR} mice (red), basophil sufficient (DT–, darker color) or basophil-depleted (DT+, lighter color) (n

=7/5/5/6). (**e**) Representative contour plots showing the CD4$^+$ effector T helper (TH) cells (non-TFH cells) gating strategy (defined as CD45$^+$ CD19$^-$ TCRβ$^+$ CD8α$^-$ CD4$^+$ CD44$^+$ CXCR5$^-$ cells) in splenocytes from a basophil-sufficient pristane-treated *Mcpt8*^{DTR} mouse. (**f**,**g**,**h**) Proportions (%) of IL-4 (**f**), IFNγ (**g**), and IL-17A (**h**) producing cells among TH cells in splenocytes stimulated with PMA and Ionomycin (P/I +) or not (P/I–) from PBS-injected mice (blue bars) and pristane-injected *Mcpt8*^{DTR} mice (red bars), basophil sufficient (DT–, darker color) or basophil-depleted (DT+, lighter color) (n =7/5/4/6). (**i**,**j**,**k**) Proportions (%) of IL-4 (**i**), IFNγ (**j**), and IL-17A (**k**) producing cells among TH cells in splenocytes stimulated with PMA and Ionomycin (P/I +) or not (P/I–) from aged *Mcpt8*^{DTR} (blue bars) or *Lyn*$^{-/-}$ *Mcpt8*^{DTR} (red bars) mice depleted in basophils (DT+, lighter color) or not (DT–, darker color) (n =4/4/4/4). (**a**-**k**) Results are from at least three independent experiments and presented as individual values in bars representing the mean values ± s.e.m. Statistical analyses were by two-way ANOVA test followed by Tukey's multiple comparisons tests between the indicated groups. *P* values are shown above each bracket. NS: not significant. SA: streptavidin. Source data are provided in the Source Data file.

Supplementary Figure 7

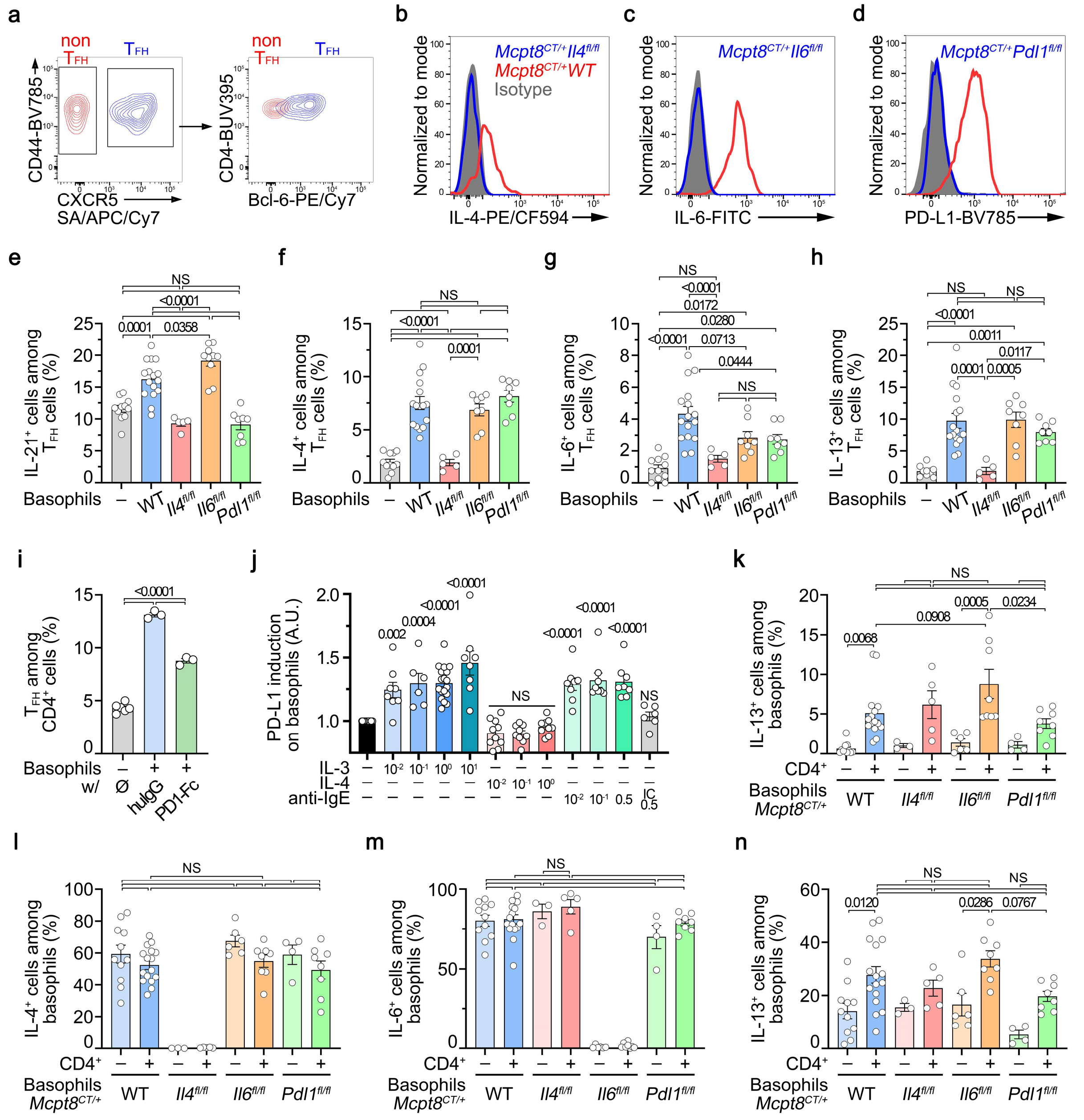

Supplementary Fig. 7: Effective CRE-mediated floxed gene deletion in basophils and PMA-Ionomycin-induced cytokine production by basophils co-cultured or not with naïve CD4$^+$ T cells

(**a**) Intracellular staining for Bcl6 expression in CD3/CD28 activated CD4$^+$CD44$^+$ PD1$^+$ T cells expressing CXCR5 (TFH cells, blue) or not (non TFH cells, red) after 3 days of culture with WT basophils as described in **Fig. 4**. (**b**) Histogram plot showing IL-4 production by PMA and ionomycin stimulated spleen basophils from *Mcpt8*$^{CT/+}$*Il4*$^{+/+}$ (WT, red) and *Mcpt8*$^{CT/+}$*Il4*$^{fl/fl}$ (IL-4 deficient basophils, blue) mice. The isotype control signal is overlaid (grey-filled). (**c**) Histogram plot showing IL-6 production by PMA and Ionomycin stimulated spleen basophils from *Mcpt8*$^{CT/+}$*Il6*$^{+/+}$ (WT, red) and *Mcpt8*$^{CT/+}$*Il6*$^{fl/fl}$ (IL-6 deficient basophils, blue) mice. The isotype control signal is overlaid (grey-filled). (**d**) Histogram plot showing PD-L1

expression on spleen basophils from *Mcpt8*$^{CT/+}$*Pdl1*$^{+/+}$ (WT, red) and *Mcpt8*$^{CT/+}$*Pdl1*$^{fl/fl}$ (PD-L1 deficient basophils, blue). The isotype control signal is overlaid (grey-filled). (**e**-**h**) Proportions (%) of IL-21- (**e**), IL-4- (**f**), IL-6- (**g**), and IL-13- (**h**) producing cells among TFH cells restimulated with PMA and ionomycin and brefeldin A for the last 4 hours of the culture from the same conditions as described in **Fig. 4a** (n= 11/16/5/8/8). (**i**) Proportions (%) of TFH among CD4^{+} T cells after three days of culture without (–, Ø; grey filled) or with WT basophils in the presence of (w/) human IgG (blue) or mouse PD-1-hIgG Fc recombinant protein (green) (n= 6/3/3). (**j**) PD-L1 expression induction on WT basophils after stimulation of whole splenocytes with the indicated doses (in ng/mL) of IL-3 (blue), IL-4 (red), or anti-IgE (green) or isotype control (rat IgG1,κ; grey) for 2 hours. PD-L1 levels were normalized on the corresponding unstimulated (NS) condition for each sample and statistics against unstimulated condition are shown above each bar (n= 18/8/6/18/8/10/9/9/8/8/8/6). (**k**) Proportions (%) of spontaneous IL-13-producing cells among basophils of the indicated genotypes cultured as described in **Fig. 5b** (n= 11/15/3/5/6/8/4/8). (**l**-**n**) Proportions (%) of PMA and ionomycin-induced IL-4- (**l**), IL-6- (**m**) and IL-13- (**n**) producing cells among basophils of the indicated genotypes cultured without (–, light colors) or with (+, dark colors) purified wild-type CD3/CD28 activated naïve CD4^{+} T cells as described in **Fig 5** (n= 11/15/3/5/6/8/4/8). (**e**-**n**) Results are from at least three independent experiments and presented as individual values in bars representing the mean values ± s.e.m. (**e**-**n**) Statistical analyses were by one way ANOVA followed by Tukey's multiple comparisons tests between the indicated groups. *P* values are shown above each bracket. NS: not significant. Source data are provided in the Source Data file.

Supplementary Figure 8

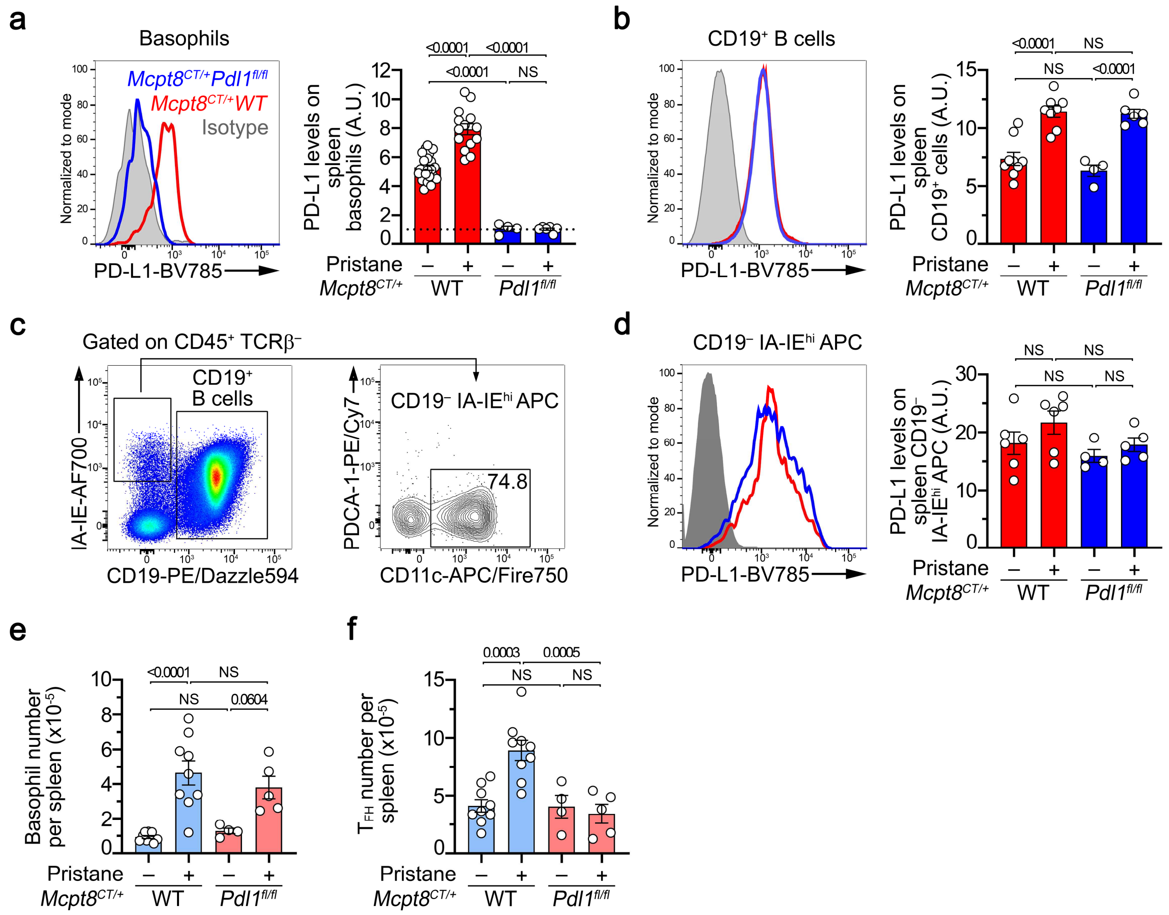

Supplementary Fig. 8: CRE-mediated *Pdl1* floxed gene deletion selectively in the basophil compartment

(**a**) **Left**. FACS histogram plot showing PD-L1 expression on spleen basophils from *Mcpt8*$^{CT/+}$*Pdl1*$^{+/+}$ (WT, red) and *Mcpt8*$^{CT/+}$*Pdl1*$^{fl/fl}$ (*Pdl1*$^{fl/fl}$, blue). The isotype control signal is overlaid (grey-filled). **Right**. PD-L1 expression quantification on spleen basophils from *Mcpt8*$^{CT/+}$ (WT) (red) or *Mcpt8*$^{CT/+}$*Pdl1*$^{fl/fl}$ (*Pdl1*$^{fl/fl}$) (blue) mice injected with PBS (–) or with pristane (+). (n= 22/14/3/5) as determined by flow cytometry. For WT mice, same data as presented in **Fig. 7a**. (**b**) **Left**. FACS histogram plot showing PD-L1 expression on spleen CD19^{+} cells from the same mice as in (**a**) gated as shown in (**c**). **Right**. PD-L1 expression quantification on spleen CD19^{+} B cells from mice as in (**a**). (n= 9/8/4/6) as determined by flow cytometry. (**c**) Gating strategy for CD19^{+} B cells and CD19$^{–}$ IA-IE^{hi} antigen presenting cells (APC) that are composed of 75% of conventional dendritic cells (**right**). (**d**) **Left**. FACS histogram plot showing PD-L1 expression on spleen CD19$^{–}$ IA-IE^{hi} APC the same mice as in (**a**) gated as shown in (**c**). **Right**. PD-L1 expression quantification on spleen CD19$^{–}$ IA-IE^{hi} APC from mice as in (**a**). (n= 6/6/4/5) as determined by flow cytometry. (**e**,**f**) Absolute numbers of basophils (**e**) and TFH cells (**f**) per spleen from mice as in (**a** and **Fig. 6a**) (n= 9/9/4/5). (**a**-**f**) Results are from at least three independent experiments and presented as individual values in bars representing the mean values ± s.e.m. Statistical analyses were by two way ANOVA followed by Tukey's multiple comparisons tests. *P* values are shown above each bracket. NS: not significant. Source data are provided in the Source Data file.

Supplementary Figure 9

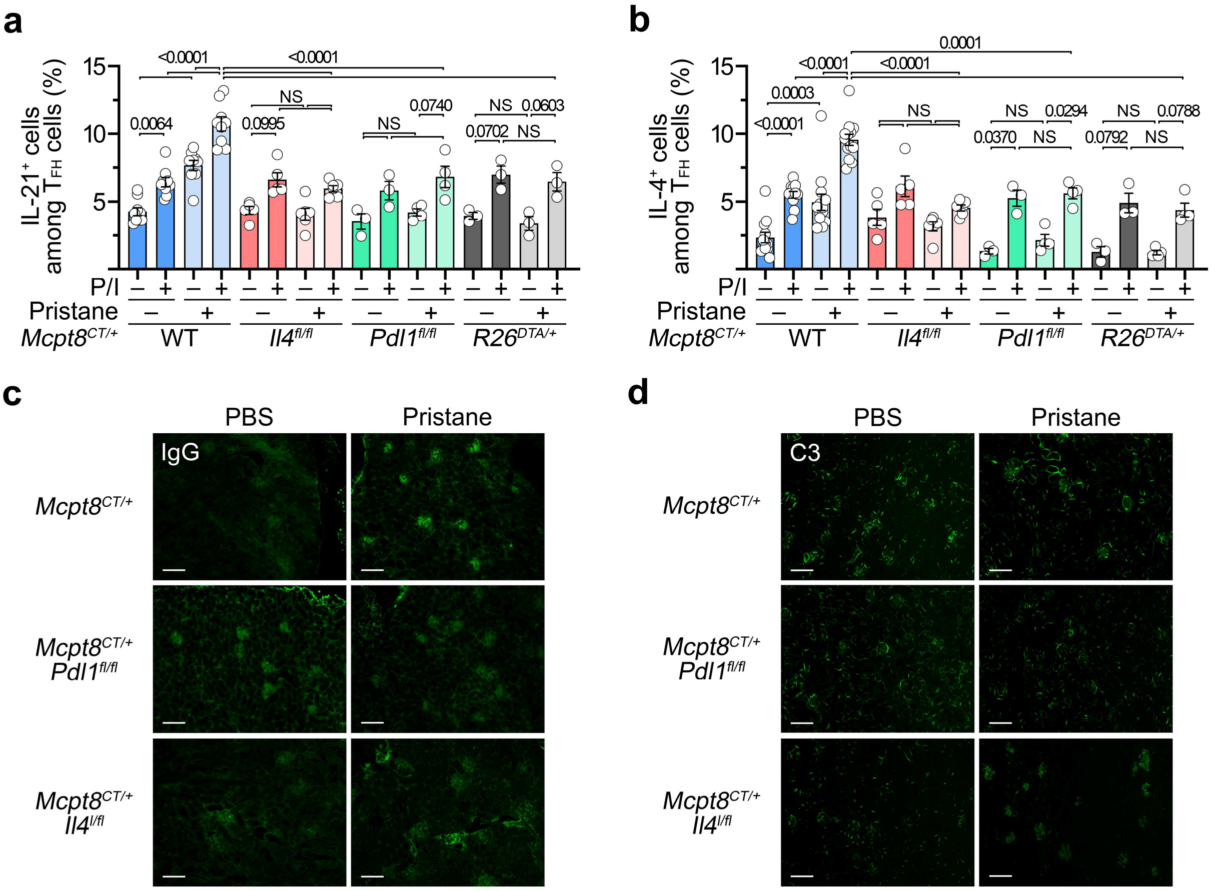

Supplementary Fig. 9: IL-21 and IL-4 production by TFH cells, IgG and C3 deposits in kidneys of PBS- or pristane-injected animals

(**a**,**b**) Splenocytes from *Mcpt8$^{CT/+}$* (WT, blue), *Mcpt8$^{CT/+}$Il4$^{fl/fl}$* (*Il4$^{fl/fl}$*, red), *Mcpt8$^{CT/+}$Pdl1$^{fl/fl}$* (*Pdl1$^{fl/fl}$*, green) and basophil-deficient (*Mcpt8$^{CT/+}$R26$^{DTA/+}$*) (*R26$^{DTA/+}$*, grey) PBS-treated (Pristane –, darker colors) or pristane-treated (Pristane +, lighter colors) mice were stimulated with PMA and Ionomycin (P/I) (+) or not (–) as described in the methods section. Intracellular flow cytometry was realized to visualize the indicated cytokine productions on the indicated cellular compartments. (**a**,**b**) Proportions (%) of IL-21 (**a**) and IL-4 (**b**) producing cells among TFH cells in splenocytes (n= 9/9/5/6/3/4/3/3). Results are presented as individual values in bars representing the mean values ± s.e.m. Statistical analyses were done by one way ANOVA followed by Tukey's multiple comparisons tests. *P* values are shown above each bracket. NS: not significant. (**c**,**d**) Representative pictures of one field of kidney from mice with the indicated genotypes treated without (PBS) or with pristane for 8 weeks showing the intensity of anti-IgG (**c**) or anti-C3 (**d**) staining by immunofluorescence. Scale bar = 200 µm. Related to **Figures 6** and **7**. Source data are provided in the Source Data file.

Supplementary Figure 10

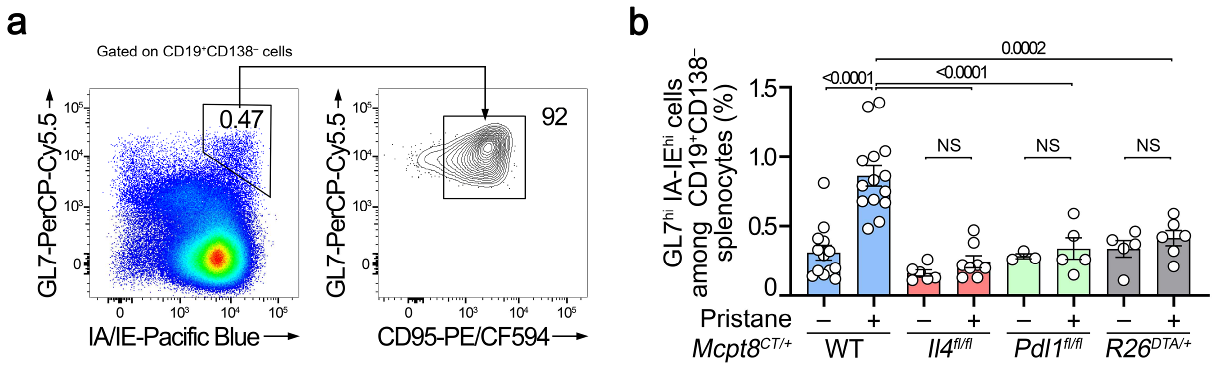

Supplementary Fig. 10: Germinal center (GC) B cell induction in pristane-treated mice

(**a**) Gating strategy for spleen CD19^{+} CD138^{-} GL7^{hi} IA-IE^{hi} B cells showing 92% identity with GC B cells gated with a conventional approach (CD19^{+} CD138^{-} GL7^{hi} CD95^{+}). (**b**) Proportions (%) of GL7^{hi} IA-IE^{hi} cells among CD19^{+} CD138^{-} B cells in splenocytes from *Mcpt8*$^{CT/+}$ (WT, blue), *Mcpt8*$^{CT/+}$*Il4*$^{fl/fl}$ (*Il4*$^{fl/fl}$, red), *Mcpt8*$^{CT/+}$*Pdl1*$^{fl/fl}$ (*Pdl1*$^{fl/fl}$, green) and basophil-deficient (*Mcpt8*$^{CT/+}$*R26*$^{DTA/+}$) (*R26*$^{DTA/+}$, grey) PBS-treated (Pristane –) or pristane-treated (Pristane +) mice (n= 13/14/6/8/3/5/5/6). Results are presented as individual values in bars representing the mean values ± s.e.m. Statistical analyses were by two way ANOVA followed by Tukey's multiple comparisons tests between the indicated groups. *P* values are shown above each bracket. NS: not significant. Source data are provided in the Source Data file.

Supplementary Figure 11

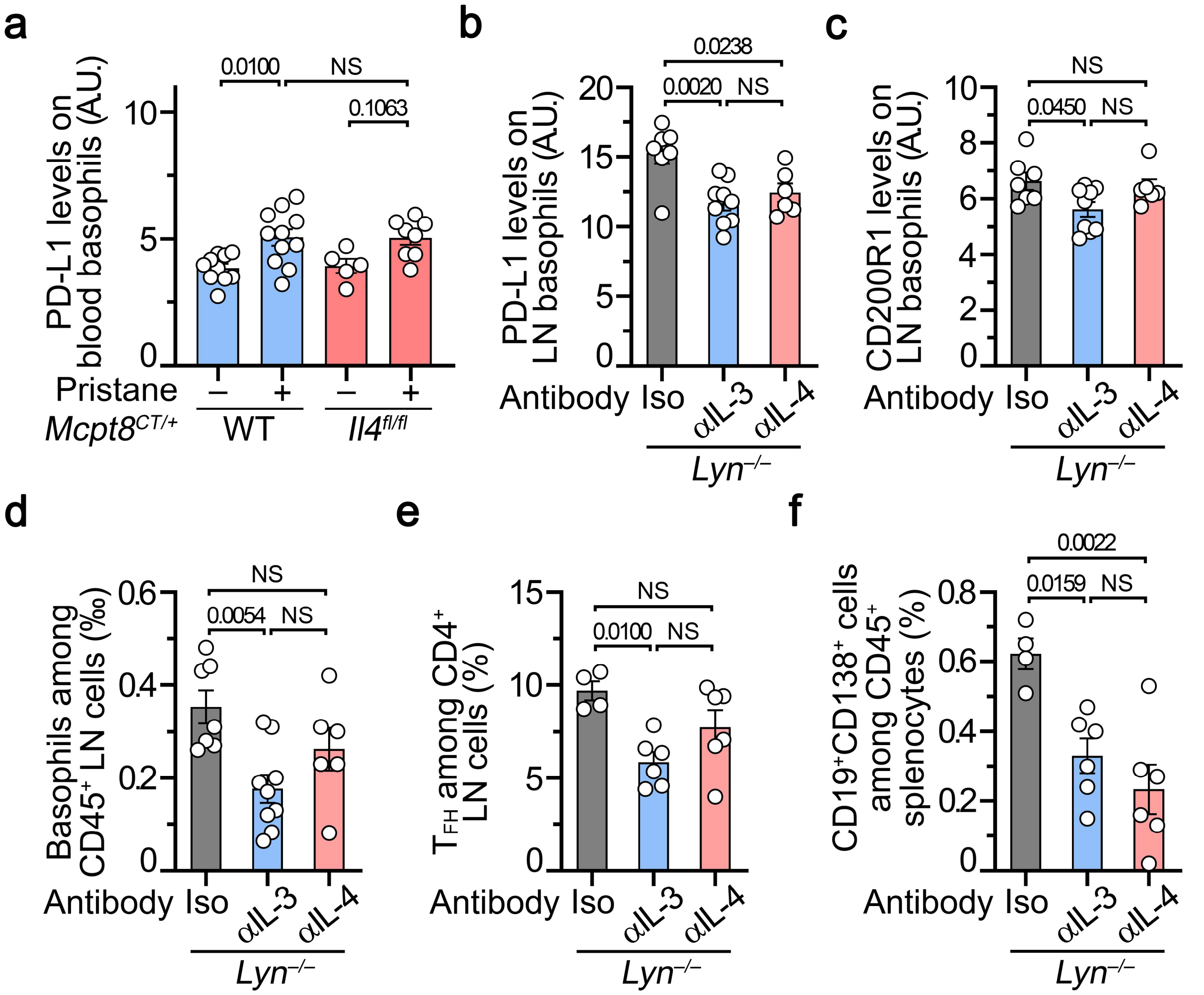

Supplementary Fig. 11: IL-4 and IL-3 control PD-L1 expression, activation and localization of basophils in the lupus-like context

(**a**) PD-L1 expression levels on blood basophils from *Mcpt8*$^{CT/+}$ (WT) (blue) or *Mcpt8*$^{CT/+}$ *Il4*$^{fl/fl}$ (*Il4*$^{fl/fl}$) (red) mice injected with PBS (–) or with pristane (+) (n= 10/11/5/8). (**b**,**c**) PD-L1 (**b**) and CD200R1 (**c**) expression levels on basophils from lymph nodes (LN) of aged *Lyn*$^{-/-}$ mice as described in **Fig. 7c** (n= 7/9/6). (**d**,**e**,**f**) Proportions (‰) of basophils among CD45^{+} LN cells (**d**) (n= 7/9/6), proportions (%) of TFH cells among LN CD4^{+} T cells (**e**) (n= 4/6/6) and CD19^{+}CD138^{+} cells among CD45^{+} splenocytes (**f**) (n= 4/6/6) from aged *Lyn*$^{-/-}$ mice as described in **Fig. 8c**. (**a**-**f**) Results are from at least two independent experiments and presented as individual values in bars representing the mean values ± s.e.m.. (**a**) Statistical analyses were by two-way ANOVA test followed by Tukey's multiple comparisons tests between the indicated groups. (**b**-**f**) Statistical analyses were by one-way ANOVA test followed by Tukey's multiple comparisons tests between the indicated groups. *P* values are shown above each bracket. NS: not significant. Source data are provided in the Source Data file.

Supplementary Figure 12

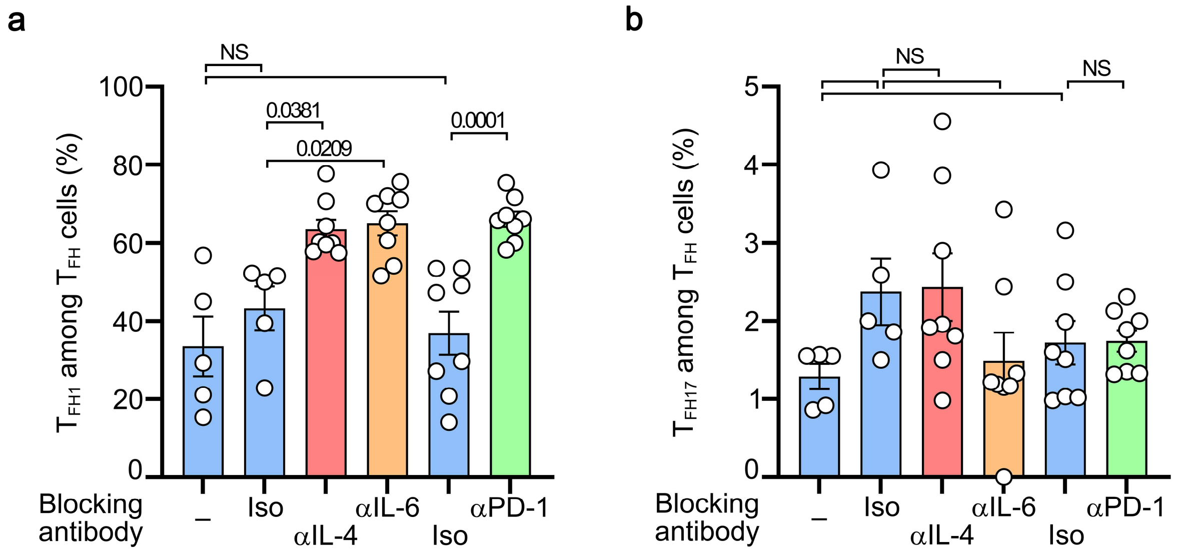

Supplementary Fig. 12: Human basophils drive TFH2 cell differentiation through IL-4, IL-6, and PD-L1 dependent mechanisms *ex vivo*

(**a**,**b**) Proportions (%) of TFH1 (**a**) and TFH17 (**b**) cells among TFH cells after culture as described in (**Fig. 9d**). TFH1 cells were defined as $CD4^+$ $PD1^+$ $CXCR5^+$ $ICOS^+$ $CCR6^-$ $CXCR3^+$ cells and TFH17 cells as $CD4^+$ $PD1^+$ $CXCR5^+$ $ICOS^+$ $CCR6^+$ $CXCR3^-$ cells (n= 5/5/8/8/8/8). (**a**,**b**) Data are presented as individual values in bars representing the mean values ± s.e.m. Results are from three independent experiments. Statistical analyses were done by one-way ANOVA test followed by Tukey's multiple comparisons tests between the indicated groups. *P* values are shown above each bracket. NS: not significant. Source data are provided in the Source Data file.

Supplementary Figure 13

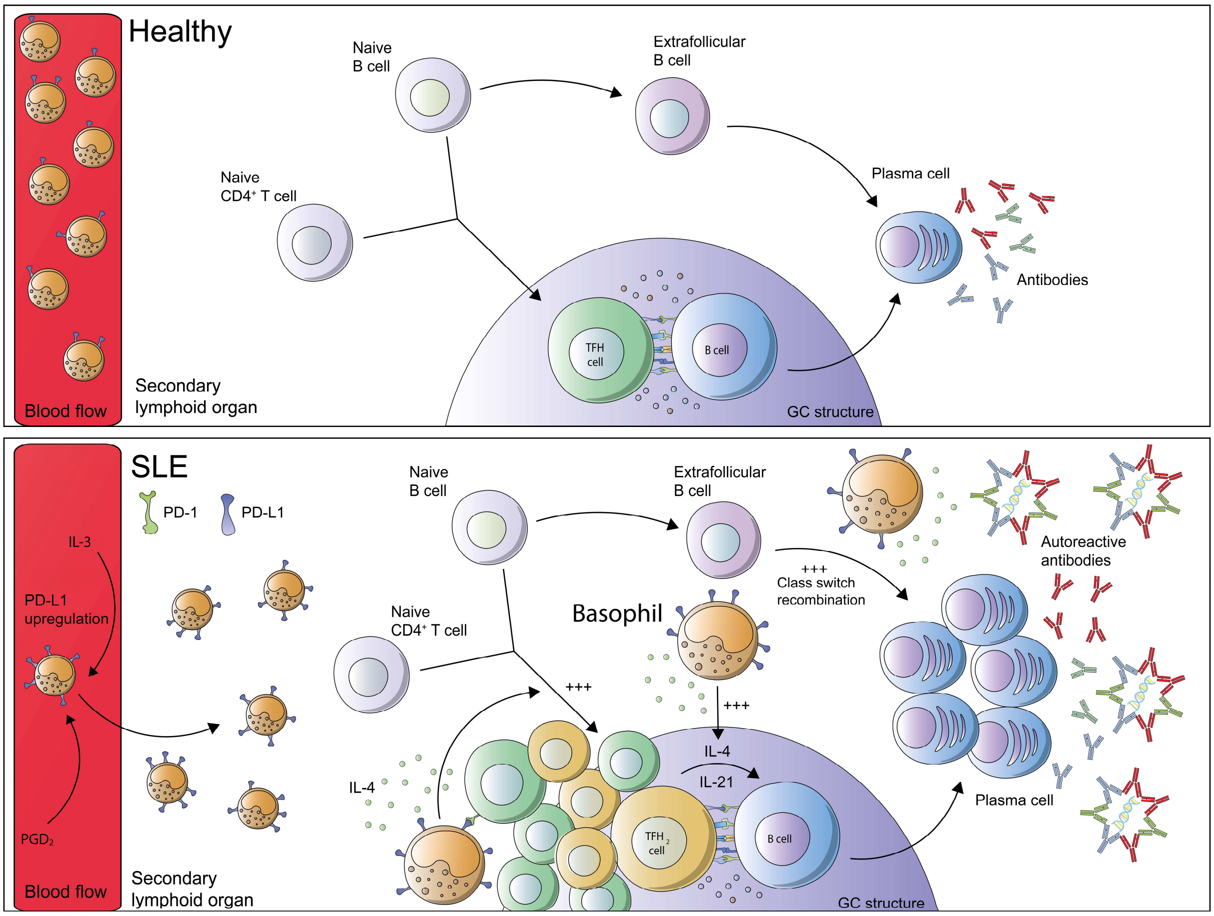

Supplementary Fig. 13: PD-L1- and IL-4-expressing basophils promote pathogenic accumulation of T follicular helper cells in lupus.

In a healthy context (top), unstimulated basophils do not leave the blood stream. In this condition, basophils do not contribute to follicular and extrafollicular responses to a given antigen in secondary lymphoid organs (SLO). In the SLE context (bottom), IL-3 upregulates PD-L1 expression by basophils and prostaglandin D_2 (PGD_2) (through CXCR4 externalization induction) enables basophil accumulation in SLO. There, in a PD-L1- and IL-4-dependent manner, basophils promote CD4+ T cell differentiation into TFH cells mainly of the TFH2 cell subset, germinal center (GC) formation, plasma cell accumulation and autoantibody production. Basophils influence also the maturation of extrafollicular B cells in a PD-L1 dependent way and class switch recombination in an IL-4 dependent manner.

Supplementary Table 1. SLE Patient and healthy control characteristics

Variables	SLE patients				Healthy controls
	ALL SLE	Inactive (SLEDAI≤1)	Mild (1<SLEDAI≤4)	Active (SLEDAI>4)	
Demographic characteristics					
n	204	54	51	99	43
Age, mean±SD, yr	38.87±12.4	44.43±13.5	39.4±11.9	35.6±10.9	34±9.2
Female, n (%)	184 (90)	47 (87)	47 (92)	90 (90)	32 (74)
Lupus characteristics					
Disease duration, mean±SD, yr	9.5±8.5	14.2±9.0	10.4±7.3	7.00±7.8	-
Anti-dsDNA Ab positive, n (%)	111 (54)	7 (12)	26 (51)	78 (79)	-
History of lupus nephritis, n (%)	163 (80)	32 (59)	37 (73)	89 (90)	-
SLEDAI					
Mean±SD	6.2±6.3	0.0±0.2	3.2±0.9	11.2±5.5	-
Median (Min-Max)	4 (0-33)	0 (0-1)	4 (2-4)	10 (5-33)	-
Treatment characteristics					
Current prednisone dose (mg/day)					
Mean±SD	13.5±38.8	3.7±3.8	7.8±7.9	22.2±54.8	-
15mg/day or higher, n (%)	39 (19)	1 (2)	8 (16)	30 (30)	-
Concurrent immuno-suppressive therapy (n, %)					
hydroxychloroquine	174 (85)	43 (80)	46 (90)	82 (83)	-
mycophenolate mofetil	67 (33)	18 (33)	22 (43)	27 (27)	-
IV cyclophosphamide	13 (6)	1 (2)	1 (2)	11 (11)	-
azathioprine	16 (8)	0 (0)	5 (10)	11 (11)	-

SD: standard deviation; yr: year; IV: intravenous.

Supplementary Table 2.

	Healthy control basophils			SLE patient basophils			
Surface Marker	Mean	SD	n	Mean	SD	n	p-value
PD-L1	5.603	2.028	39	8.455	4.308	204	<0.0001
PD-L2	0.9547	0.1361	5	1.085	0.2912	42	0.2737
ICOSL	1.676	0.2078	5	1.757	0.5207	56	0.9492
OX40L	2.499	1.842	18	2.647	1.92	178	0.8469
CD84	21.41	4.981	20	26.09	11.2	74	0.0956

Supplementary Table 2: Expression levels of some T cell interacting surface molecules on basophils from healthy controls and SLE patients

Expression levels on basophils of the indicated surface markers were determined by flow cytometry as described in supplementary Fig. S1 and in the Methods section. “Mean” corresponds to the mean of the ratio of the geometric mean fluorescence intensity (gMFI) of the indicated surface marker and the gMFI of the isotype control as determined by flow cytometry for each group. SD: standard deviation. Statistical analyses were done by two-sided Mann-Whitney U tests between the two groups.

Supplementary Table 3 : Resource Table

REAGENT or RESOURCE	SOURCE	IDENTIFIER	Concentration used (for FACS staining: 200 µL for 2x10^6 cells)
Antibodies			
Alexa Fluor 488 anti-mouse Complement Component C3 (Clone: 11H9)	Santa Cruz Biotechnology	(Cat# sc-58926, RRID:AB_1119819)	2 µg/mL
Alexa Fluor 488 anti-mouse/human CD44 (Clone: IM7)	Biolegend	(Cat# 103016, RRID:AB_493679)	1 µg/mL
Alexa Fluor 488 anti-mouse IgG Fcγ fragment specific (Goat Polyclonal)	Jackson ImmunoResearch	(Cat# 115-545-008, RRID:AB_2338842)	20 µg/mL
Alexa Fluor 488 anti-mouse/human CD45R/B220 Antibody (Clone: RA3-6B2)	Biolegend	(Cat# 103225, RRID:AB_389308)	10 µg/mL
Alexa Fluor 488 anti-mouse CD25 (Clone: PC61)	Biolegend	(Cat# 102017, RRID:AB_493333)	2.5 µg/mL
Alexa Fluor 488 anti-mouse IgD Antibody (Clone: 11-26c.2a)	Biolegend	(Cat# 405718, RRID:AB_10730619)	2.5 µg/mL
Alexa Fluor 647 anti-mouse FcεRIα (Clone: MAR-1)	Biolegend	(Cat# 134310, RRID:AB_1626093)	2.5 µg/mL
Alexa Fluor 647 anti-mouse CD3ε (Clone: 145-2C11)	Biolegend	(Cat# 100322, RRID:AB_389322)	2.5 µg/mL
Alexa Fluor 647 anti-mouse IgG (Clone: Poly4053)	Biolegend	(Cat# 405322, RRID:AB_2563045)	2.5 µg/mL
Alexa Fluor 647 anti-mouse/human GL7 Antigen Antibody (Clone: GL7)	Biolegend	(Cat# 144606, RRID:AB_2562185)	10 µg/mL
Alexa Fluor 700 anti-mouse CD45 (Clone: 30-F11)	Biolegend	(Cat# 103128, RRID:AB_493715)	1 µg/mL
Alexa Fluor 700 anti-mouse CD62L (Clone: MEL-14)	Biolegend	(Cat# 104426, RRID:AB_493719)	2.5 µg/mL
Alexa Fluor 700 anti-mouse Ly-6G/Ly-6C Antibody (Clone: Gr-1)	Biolegend	(Cat# 108422, RRID:AB_2137487)	0.5 µg/mL
Alexa Fluor 700 anti-mouse I-A/I-E Antibody (Clone: M5/114.15.2)	Biolegend	(Cat# 107622, RRID:AB_493727)	1 µg/mL
APC anti-mouse IL-3 Antibody (Clone: MP2-8F8)	Biolegend	(Cat# 503908, RRID: AB_2810628)	2 µg/mL
APC/Fire 750 anti-mouse CD4 (Clone: RM4-5)	Biolegend	(Cat# 100568, RRID:AB_2629699)	1 µg/mL
APC/Cyanine7 anti-mouse CD4 (Clone: RM4-5)	Biolegend	(Cat# 100526, RRID:AB_312727)	1 µg/mL
APC/Cyanine7 anti-mouse TCR β chain (Clone: H57-597)	Biolegend	(Cat# 109220, RRID:AB_893624)	1 µg/mL
APC/Fire 750 anti-mouse TCR β chain (Clone: H57-597)	Biolegend	(Cat# 109246, RRID:AB_2629697)	1 µg/mL
APC/Cyanine7 anti-mouse CD19 (Clone: 6D5)	Biolegend	(Cat# 115530, RRID:AB_830707)	1 µg/mL
APC/Fire 750 anti-mouse CD19 (Clone: 6D5)	Biolegend	(Cat# 115558, RRID:AB_2572120)	1 µg/mL
APC/Cyanine7 anti-mouse CD117 (c-kit) (Clone: 2B8)	Biolegend	(Cat# 105826, RRID:AB_1626278)	1 µg/mL

APC/Fire 750 anti-mouse CD117 (c-Kit) (Clone: 2B8)	Biolegend	(Cat# 105838, RRID:AB_2616739)	1 μg/mL
APC/Fire 750 anti-mouse CD11c Antibody (Clone: N418)	Biolegend	(Cat# 117352, RRID:AB_2572124)	1 μg/mL
Biotin anti-mouse CD185 (CXCR5) (Clone: L138D7)	Biolegend	(Cat# 145510, RRID:AB_2562126)	5 μg/mL
Biotin anti-mouse CD4 (Clone: RM4-5)	Biolegend	(Cat# 100508, RRID:AB_312711)	5 μg/mL
Biotin anti-mouse CD19 (Clone: 6D5)	Biolegend	(Cat# 115504, RRID:AB_313639)	5 μg/mL
Biotin anti-mouse CD8a (Clone: 53-6.7)	Biolegend	(Cat# 100704, RRID:AB_312743)	5 μg/mL
Biotin anti-mouse NK-1.1 (Clone: PK136)	Biolegend	(Cat# 108704, RRID:AB_313391)	5 μg/mL
BUV395 anti-mouse CD45 (Clone: 30-F11)	BD Biosciences	(Cat# 564279, RRID:AB_2651134)	0.5 μg/mL
Brilliant Violet 421 anti-mouse CD4 (Clone: GK1.5)	Biolegend	(Cat# 100438, RRID:AB_11203718)	0.12 μg/mL
Brilliant Violet 421 anti-mouse CD4 (Clone : RM4-5)	Biolegend	(Cat# 100563, RRID:AB_2563052)	10 μg/mL (IF)
Brilliant Violet 421 anti-mouse CD279 (PD-1) (Clone: 29F.1A12)	Biolegend	(Cat# 135218, RRID:AB_2561447)	0.12 μg/mL
Brilliant Violet 421 anti-mouse CD138 (Syndecan-1) (Clone: 281-2)	Biolegend	(Cat# 142508, RRID:AB_11203544)	0.25 μg/mL
Brilliant Violet 421 anti-mouse/human CD44 (Clone: IM7)	Biolegend	(Cat# 103040, RRID:AB_2616903)	0.25 μg/mL
Brilliant Violet 605 anti-mouse CD45 (Clone: 30-F11)	Biolegend	(Cat# 103140, RRID:AB_2562342)	0.2 μg/mL
Brilliant Violet 605 anti-mouse CD279 (PD-1) (Clone: 29F.1A12)	Biolegend	(Cat# 135220, RRID:AB_2562616)	1 μg/mL
Brilliant Violet 605 anti-mouse CD152 (Clone: UC10-4B9)	Biolegend	(Cat# 106323, RRID:AB_2566467)	1 μg/mL
Brilliant Violet 785 anti-mouse CD19 (Clone: 6D5)	Biolegend	(Cat# 115543, RRID:AB_11218994)	0.15 μg/mL
Brilliant Violet 785 anti-mouse/human CD44 (Clone: IM7)	Biolegend	(Cat# 103059, RRID:AB_2571953)	0.5 μg/mL
Brilliant Violet 785 anti-mouse CD274 (B7-H1, PD-L1) (Clone: 10F.9G2)	Biolegend	(Cat# 124331, RRID:AB_2629659)	1 μg/mL
eFluor 450 anti-mouse IL-6 (Clone: MP5-20F3)	Thermo Fisher Scientific	(Cat# 48-7061-82, RRID:AB_2574103)	2 μg/mL
eFluor 450 anti-mouse IL-21 (Clone: FFA21)	Thermo Fisher Scientific	(Cat# 48-7211-82, RRID:AB_2811832)	2 μg/mL
FITC anti-mouse Complement Component C3 (Clone: RmC11H9)	Cedarlane	(Cat# CL7503F, RRID:AB_10061294)	10 μg/mL
FITC anti-mouse IgM (Goat polyclonal)	BioRad (AbD Serotec)	(Cat# 102002, RRID:AB_619870)	2.5 μg/mL
FITC anti-mouse CD123 (Clone: 5B11)	Thermo Fisher Scientific	(Cat# 11-1231-82, RRID:AB_465192)	2.5 μg/mL
FITC anti-mouse CD49b (Clone: HMα2)	Biolegend	(Cat# 103504, RRID:AB_313027)	2.5 μg/mL
FITC anti-mouse IL-6 (Clone: MP5-20F3)	Thermo Fisher Scientific	(Cat# 11-7061-82, RRID:AB_465394)	2.5 μg/mL

FITC anti-mouse TNF-α (Clone: MP6-XT22)	R&D Systems	(Cat# IC410F, RRID:AB_357323)	2.5 µg/mL
FITC anti-mouse IgA (Polyclonal IgG)	BioRad (AbD Serotec)	(Cat# STAR137, RRID:AB_2075638)	2.5 µg/mL
FITC anti-mouse IgE (Clone: RME-1)	Biolegend	(Cat# 406906, RRID:AB_493289)	10 µg/mL (IF)
Pacific Blue anti-mouse CD49b (pan-NK cells) (Clone: DX5)	Biolegend	(Cat# 108918, RRID:AB_2265144)	2.5 µg/mL
Pacific Blue anti-mouse I-A/I-E Antibody (Clone: M5/114.15.2)	Biolegend	(Cat# 107620, RRID:AB_493527)	2.5 µg/mL
PE anti-mouse IL-21 (Clone: FFA21)	Thermo Fisher Scientific	(Cat# 12-7211-82, RRID:AB_1834466)	2 µg/mL
PE anti-mouse CD278 (ICOS) (Clone: 15F9)	Biolegend	(Cat# 107706, RRID:AB_313335)	2 µg/mL
PE anti-mouse FOXP3 (Clone: FJK-16s)	Thermo Fisher Scientific	(Cat# 12-5773-82, RRID:AB_465936)	4 µg/mL
PE anti-mouse CD200R3 (Clone: Ba13)	Biolegend	(Cat# 142206, RRID:AB_10916520)	0.5 µg/mL
PE-CF594 anti-mouse IL-4 (Clone: 11B11)	BD Biosciences	(Cat# 562450, RRID:AB_2737616)	2 µg/mL
PE-CF594 Hamster Anti-Mouse CD95 (Clone: Jo2)	BD Biosciences	(Cat# 562499, RRID:AB_11154214)	0.5 µg/mL
PE/Cyanine7 anti-mouse CD200R3 (Clone: Ba13)	Biolegend	(Cat# 142212, RRID:AB_2814046)	0.5 µg/mL
PE/Cyanine7 anti-mouse CD317 (BST2, PDCA-1) (Clone: eBio927)	Thermo Fisher Scientific	(Cat# 25-3172-82, RRIDAB_2573440)	1 µg/mL
PE/Cyanine7 anti-human/mouse Bcl-6 Antibody (Clone: 7D1)	Biolegend	(Cat# 358512, RRID:AB_2566196)	5 µg/mL
PE/Cyanine7 anti-mouse CD45 (Clone: 30-F11)	Biolegend	(Cat# 103114, RRID:AB_312979)	0.4 µg/mL
PE/Cyanine7 anti-mouse IL-13 (Clone: eBio13A)	Thermo Fisher Scientific	(Cat# 25-7133-82, RRID:AB_2573530)	2 µg/mL
PE/Cyanine7 anti-mouse CD62L (Clone: MEL-14)	Biolegend	(Cat# 104418, RRID:AB_313103)	1 µg/mL
PE/Cyanine7 anti-mouse CD274 (B7-H1, PD-L1) (Clone: 10F.9G2)	Biolegend	(Cat# 124314, RRID:AB_10643573)	1 µg/mL
PE/Cyanine7 anti-mouse CD90.2 (Thy-1.2) Antibody (Clone: 53-2.1)	Biolegend	(Cat# 140310, RRID:AB_10643586)	1 µg/mL
PE/Dazzle 594 anti-mouse CD279 (PD-1) (Clone: 29F.1A12)	Biolegend	(Cat# 135228, RRID:AB_2566006)	1 µg/mL
PE/Dazzle 594 anti-mouse CD19 (Clone: 6D5)	Biolegend	(Cat# 115554, RRID:AB_2564001)	1 µg/mL
PE/Dazzle 594 anti-mouse/human CD44 (Clone: IM7)	Biolegend	(Cat#103056, RRID:AB_2564044)	0.4 µg/mL
PE/Dazzle 594 anti-mouse/human CD11b Antibody (Clone: M1/70)	Biolegend	(Cat#101256, RRID:AB_2563648)	0.5 µg/mL
PerCP/Cyanine5.5 anti-mouse CD8a (Clone: 53-6.7)	Biolegend	(Cat# 100734, RRID:AB_2075238)	1 µg/mL
PerCP/Cyanine5.5 anti-mouse CD4 (Clone: RM4-5)	Biolegend	(Cat# 100540, RRID:AB_893326)	1 µg/mL
PerCP/Cyanine5.5 anti-mouse IFN-γ (Clone: XMG1.2)	Biolegend	(Cat# 505822, RRID:AB_961359)	1 µg/mL

PerCP/Cyanine5.5 anti-mouse/ human GL7 Antigen Antibody (Clone: GL7)	Biolegend	(Cat# 144610, RRID:AB_2562979)	1 μg/mL
PerCP/eFluor710 anti-mouse IgM (Clone: II/41)	Thermo Fisher Scientific	(Cat#46-5790-82, RRID:AB_1834435)	1 μg/mL
PerCP/eFluor710 anti-mouse CD200R (Clone: OX110)	Thermo Fisher Scientific	(Cat#46-5201-82, RRID: AB_10804765)	1 μg/mL
Alexa Fluor 647 anti-human CD294 (CRTH2) (Clone: BM16)	Biolegend	(Cat# 350104, RRID:AB_10642025)	1 μg/mL
Alexa Fluor 647 anti-human CD273 (B7-DC, PD-L2) (Clone: MIH18)	Biolegend	(Cat# 345514, RRID:AB_2728313)	0.5 μg/mL
Alexa Fluor 700 anti-human/mouse/rat CD278 (ICOS) (Clone: C398.4A)	Biolegend	(Cat# 313528, RRID:AB_2566126)	5 μg/mL
APC anti-human CD4 (Clone: RPA-T4)	Biolegend	(Cat# 300537, RRID:AB_2562051)	1 μg/mL
APC anti-human CD275 (B7-H2, ICOSL) (Clone: 2D3)	Biolegend	(Cat# 309408, RRID:AB_2565557)	1 μg/mL
APC/Cyanine7 anti-human CD183 (CXCR3) (Clone: G025H7)	Biolegend	(Cat# 353722, RRID:AB_2561423)	1 μg/mL
Brilliant Violet 421 anti-human CD203c (E-NPP3) (Clone: NP4D6)	Biolegend	(Cat# 324612, RRID:AB_2563848)	0.12 μg/mL
Brilliant Violet 421 anti-human CD274 (B7-H1, PD-L1) (Clone: 29E.2A3)	Biolegend	(Cat# 329714, RRID:AB_2563852)	0.25 μg/mL
Brilliant Violet 421 anti-human CD279 (PD-1) (Clone: NAT105)	Biolegend	(Cat# 367422, RRID:AB_2721517)	0.25 μg/mL
Brilliant Violet 605 anti-human CD193 (CCR3) (Clone: 5E8)	Biolegend	(Cat# 310716, RRID:AB_2563831)	0.5 μg/mL
Brilliant Violet 785 anti-human CD196 (CCR6) (Clone: G034E3)	Biolegend	(Cat# 353422, RRID:AB_2563660)	0.8 μg/mL
BUV395 anti-human CD3 (Clone: SK7 (also known as Leu-4))	BD Biosciences	(Cat# 564000, RRID:AB_2744382)	0.5 μg/mL
BUV395 anti-human CD14 (Clone: MϕP9 (also known as MϕP-9))	BD Biosciences	(Cat# 563561, RRID:AB_2744288)	1 μg/mL
BUV395 anti-human CD56 (Clone: NCAM16.2 (also known as NCAM 16))	BD Biosciences	(Cat# 563554, RRID:AB_2687886)	1 μg/mL
BUV395 anti-human CD19 (Clone: SJ25C1 (also known as SJ25-C1))	BD Biosciences	(Cat# 563551, RRID:AB_2738274)	0.5 μg/mL
PE anti-human CD185 (CXCR5) (Clone: J252D4)	Biolegend	(Cat# 356904, RRID:AB_2561813)	0.5 μg/mL
PE anti-human CD84 (Clone: CD84.1.21)	Biolegend	(Cat# 326008, RRID:AB_2229003)	1 μg/mL
PE anti-human CD252 (OX40L) (Clone: 11C3.1)	Biolegend	(Cat# 326308, RRID:AB_2207271)	4 μg/mL
PE/Cyanine7 anti-human FcεRIα (Clone: AER-37 (CRA-1))	Biolegend	(Cat# 334620, RRID:AB_10575314)	0.5 μg/mL
PE/Dazzle 594 anti-human CD123 (Clone: 6H6)	Biolegend	(Cat# 306034, RRID:AB_2566450)	0.06 μg/mL
PerCP/Cyanine5.5 anti-human CD184 (CXCR4) Antibody (Clone: 12G5)	Biolegend	(Cat# 306516, RRID:AB_10642818)	2 μg/mL

Alexa Fluor 488 Rat IgG2a, κ Isotype Control (Clone: RTK2758)	Biolegend	(Cat# 400525, RRID:AB_2864283)	2 μg/mL
Alexa Fluor 488 Goat IgG whole molecule (Goat polyclonal)	Jackson ImmunoResearch	(Cat# 005-540-003, RRID:AB_2337013)	20 μg/mL
Alexa Fluor 488 Rat IgG2b, κ Isotype Control (Clone: RTK4530)	Biolegend	(Cat# 400625, RRID:AB_389321)	1 μg/mL
Alexa Fluor 647 Armenian Hamster IgG Isotype Control (Clone: HTK888)	Biolegend	(Cat# 400924, RRID:AB_2922967)	2.5 μg/mL
Alexa Fluor 647 Rat IgG2a, κ Isotype Control (Clone: RTK2758)	Biolegend	(Cat# 400526, RRID:AB_2864284)	1 μg/mL
Alexa Fluor 647 Mouse IgG1, κ Isotype Control (Clone: MOPC-21)	Biolegend	(Cat# 400130, RRID:AB_2800436)	1 μg/mL
Alexa Fluor 647 Rat IgM, κ Isotype Control (Clone: RTK2118)	Biolegend	(Cat# 400813, RRID:AB_2086804)	10 μg/mL
Alexa Fluor 700 Rat IgG2a, κ Isotype Control (Clone: RTK2758)	Biolegend	(Cat# 400528, RRID:AB_2923249)	2.5 μg/mL
Alexa Fluor 700 Rat IgG2b, κ Isotype Control (Clone: RTK4530)	Biolegend	(Cat# 400628, RRID:AB_493783)	1 μg/mL
Alexa Fluor 700 Mouse IgG1, κ Isotype Control (Clone: MOPC-21)	Biolegend	(Cat# 400143, RRID:AB_2923250)	1 μg/mL
APC Mouse IgG1, κ Isotype Control (Clone: MOPC-21)	Biolegend	(Cat# 400119, RRID:AB_2888687)	1 μg/mL
APC Mouse IgG2b, κ Isotype Control (Clone: MPC-11)	Biolegend	(Cat# 400322, RRID:AB_326500)	1 μg/mL
APC Rat IgG1, κ Isotype Ctrl Antibody (Clone: RTK2071)	Biolegend	(Cat# 400412, RRID:AB_326518)	2 μg/mL
APC/Cyanine7 Armenian Hamster IgG Isotype Control (Clone: HTK888)	Biolegend	(Cat# 400927, RRID:AB_2923251)	1 μg/mL
APC/Cyanine7 Rat IgG2a, κ Isotype Control (Clone: RTK2758)	Biolegend	(Cat# 400523, RRID:AB_2923252)	1 μg/mL
APC/Cyanine7 Rat IgG2b, κ Isotype Control (Clone: RTK4530)	Biolegend	(Cat# 400623, RRID:AB_326565)	1 μg/mL
APC/Cyanine7 Mouse IgG1, κ Isotype Control (Clone: MOPC-21)	Biolegend	(Cat# 400127, RRID:AB_2892538)	1 μg/mL
APC/Fire 750 Armenian Hamster IgG Isotype Control (Clone: HTK888)	Biolegend	(Cat# 400961, RRID:AB_2923253)	1 μg/mL
APC/Fire 750 Rat IgG2a, κ Isotype Control (Clone: RTK2758)	Biolegend	(Cat# 400567, RRID:AB_2923254)	1 μg/mL
APC/Fire 750 Rat IgG2b, κ Isotype Control (Clone: RTK4530)	Biolegend	(Cat# 400669, RRID:AB_2905475)	1 μg/mL
Biotin Rat IgG2b, κ Isotype Control (Clone: RTK4530)	Biolegend	(Cat# 400603, RRID:AB_326547)	5 μg/mL
BUV395 Rat IgG2b, κ Isotype Control (Clone: R35-38)	BD Biosciences	(Cat# 563560, RRID:AB_2869507)	0.5 μg/mL
BUV395 Mouse IgG1, k Isotype Control (Clone: X-40)	BD Biosciences	(Cat# 563547, RRID:AB_2869503)	1 μg/mL
BUV395 Mouse IgG2b, κ Isotype Control (Clone: 27-35)	BD Biosciences	(Cat# 563558, RRID:AB_2869505)	0.5 μg/mL
Brilliant Violet 421 Rat IgG2a, κ Isotype Control (Clone: RTK2758)	Biolegend	(Cat# 400535, RRID:AB_10933427)	0.12 μg/mL

Brilliant Violet 421™ Rat IgG2b, κ Isotype Control (Clone: RTK4530)	Biolegend	(Cat# 400639, RRID:AB_10895758)	0.12 µg/mL
Brilliant Violet 421 Mouse IgG1, κ Isotype Control (Clone: MOPC-21)	Biolegend	(Cat# 400157, RRID:AB_10897939)	0.12 µg/mL
Brilliant Violet 421 Mouse IgG2b, κ Isotype Control (Clone: MPC-11)	Biolegend	(Cat# 400341, RRID:AB_10898160)	0.25 µg/mL
Brilliant Violet 605 Armenian Hamster IgG Isotype Control (Clone: HTK888)	Biolegend	(Cat# 400943, RRID:AB_2923255)	1 µg/mL
Brilliant Violet 605 Rat IgG2a, κ Isotype Control (Clone: RTK2758)	Biolegend	(Cat# 400539, RRID:AB_11126979)	1 µg/mL
Brilliant Violet 605 Rat IgG2b, κ Isotype Control (Clone : RTK4530)	Biolegend	(Cat# 400649, RRID:AB_2864282)	0.2 µg/mL
Brilliant Violet 785 Rat IgG2a, κ Isotype Control (Clone: RTK2758)	Biolegend	(Cat# 400545, RRID:AB_11218993)	1 µg/mL
Brilliant Violet 785 Rat IgG2b, κ Isotype Control (Clone: RTK4530)	Biolegend	(Cat# 400647, RRID:AB_2923256)	1 µg/mL
Brilliant Violet 785 Mouse IgG2a, κ Isotype Control (Clone: MOPC-173)	Biolegend	(Cat# 400273, RRID:AB_2923257)	0.8 µg/mL
eFluor 450 Rat IgG1, κ Isotype Control (Clone: eBRG1)	Thermo Fisher Scientific	(Cat# 48-4301-82, RRID:AB_1271984)	2 µg/mL
eFluor 450 Rat IgG2a, κ Isotype Control (Clone: eBRG1)	Thermo Fisher Scientific	(Cat# 48-4321-82, RRID:AB_1271999)	2 µg/mL
FITC Armenian Hamster IgG Isotype Control (Clone: HTK888	Biolegend	(Cat# 400905, RRID:AB_2923258)	2.5 µg/mL
FITC Rat IgG1, κ Isotype Control (Clone: RTK2071)	Biolegend	(Cat# 400405, RRID:AB_326511)	2.5 µg/mL
FITC Rat IgG2a, κ Isotype Control (Clone: RTK2758)	Biolegend	(Cat# 400506, RRID:AB_2736919)	2.5 µg/mL
Pacific Blue Rat IgM, κ Isotype Control (Clone: RTK2118)	Biolegend	(Cat# 400816, RRID:AB_10644001)	2.5 µg/mL
Pacific Blue Rat IgG2b, κ Isotype Ctrl Antibody (Clone: RTK4530)	Biolegend	(Cat# 400627, RRID:AB_493561)	2.5 µg/mL
PE Mouse IgG1, κ Isotype Control (Clone: MOPC-21)	Biolegend	(Cat# 400112, RRID:AB_2847829)	0.5 µg/mL
PE Mouse IgG2a, κ Isotype Control Antibody (Clone: MOPC-173)	Biolegend	(Cat# 400212, RRID:AB_326460)	1 µg/mL
PE Rat IgG2a, κ Isotype Control Antibody (Clone: RTK2758)	Biolegend	(Cat# 400508, RRID:AB_326530)	0.5 µg/mL
PE-CF594 Rat IgG1, κ Isotype Control (Clone: R3-34)	BD Biosciences	(Cat# 562309, RRID:AB_11153318)	2 µg/mL
PE-CF594 Hamster IgG2, λ1 Isotype Control (Clone: HIg2l1)	BD Biosciences	(Cat# 562522, RRID:AB_11153495)	0.5 µg/mL
PE/Cyanine7 Rat IgG1, κ Isotype Control (Clone: RTK2071)	Biolegend	(Cat# 400416, RRID:AB_326522)	1 µg/mL
PE/Cyanine7 Rat IgG2a, κ Isotype Control (Clone: RTK275)	Biolegend	(Cat# 400522, RRID:AB_326542)	1 µg/mL
PE/Cyanine7 Rat IgG2b, κ Isotype Control (Clone: RTK4530)	Biolegend	(Cat# 400618, RRID:AB_326560)	0.4 µg/mL
PE/Cyanine7 Mouse IgG2b, κ Isotype Control (Clone: MPC-11)	Biolegend	(Cat# 400325, RRID:AB_2923259)	0.5 µg/mL
PE/Dazzle 594 Rat IgG2a, κ Isotype Control (Clone: RTK2758)	Biolegend	(Cat# 400557, RRID:AB_2923260)	1 µg/mL

PE/Dazzle 594 Rat IgG2b, κ Isotype Control (Clone: RTK4530)	Biolegend	(Cat# 400660, RRID: AB_3096021)	0.5 µg/mL
PE/Dazzle 594 Mouse IgG1, κ Isotype Control (Clone: MOPC-21)	Biolegend	(Cat# 400175, RRID:AB_2923261)	0.06 µg/mL
PerCP/Cyanine5.5 Rat IgG2a, κ Isotype Control (Clone: RTK2758)	Biolegend	(Cat# 400531, RRID:AB_2864286)	1 µg/mL
PerCP/Cyanine5.5 Rat IgG1, κ Isotype Control (Clone: RTK2071)	Biolegend	(Cat# 400425, RRID:AB_893689)	1 µg/mL
PerCP/Cyanine5.5 Rat IgM, κ Isotype Ctrl Antibody (Clone: RTK2118)	Biolegend	(Cat# 400815, RRID:AB_2230100)	1 µg/mL
PerCP/Cyanine5.5 Mouse IgG2a, κ Isotype Ctrl Antibody (Clone: MOPC-173)	Biolegend	(Cat# 400258, RRID:AB_893689)	2 µg/mL
Anti-Mouse IgG (H+L) Cross-Adsorbed Secondary Antibody HRP coupled (Polyclonal)	Thermo Fisher Scientific	(Cat# G-21040, RRID:AB_2536527)	0.1 µg/mL
Anti-Mouse IgM Heavy Chain Antibody HRP Conjugated (Polyclonal)	Bethyl Laboratories	Cat# A90-101P, RRID:AB_67189)	0.1 µg/mL
Anti-mouse CD16/CD32 (Clone: 2.4G2)	BioXCell	(Cat# BE0307, RRID:AB_2736987)	10 µg/mL
Anti-mouse CD3ε F(ab')2 fragment (Clone: 145-2C11)	BioXCell	(Cat# BE0001-1FAB, RRID:AB_2687679)	0.5 µg/mL
Anti-mouse CD28 (Clone: PV-1)	BioXCell	(Cat# BE0015-5, RRID:AB_1107628)	0.5 µg/mL
Anti-human CD3 (Clone: OKT3)	Thermo Fisher Scientific	(Cat# 16-0037-81, RRID:AB_468854)	5 µg/mL
Anti-human/monkey CD28 (Clone: CD28.2)	BioXCell	(Cat# BE0291, RRID:AB_2687814)	2 µg/mL
Purified Anti-mouse CD279 (PD-1) (Clone: 29F.1A12)	Biolegend	(Cat# 135202, RRID:AB_1877121)	10 µg/mL
Purified Anti-human CD279 (PD-1) (Clone: EH12.2H7)	Biolegend	(Cat# 329902, RRID:AB_940488)	10 µg/mL
Purified Mouse IgG1, κ Isotype Control (Clone: MOPC-21)	Biolegend	(Cat# 400102, RRID:AB_2891079)	10 µg/mL
Purified Anti-human IL-6 (Clone: MQ2-39C3)	Biolegend	(Cat# 501204, RRID:AB_2296206)	5 µg/mL
Purified Anti-human IL-4 Antibody (Clone: MP4-25D2)	Biolegend	(Cat# 500802, RRID:AB_315121)	5 µg/mL
Purified Rat IgG1, κ Isotype Control (Clone: RTK2071)	Biolegend	(Cat# 400402, RRID:AB_326508)	5 µg/mL
Purified anti-mMCP-8 Antibody	Biolegend	(Cat# 647402, RRID: AB_2290790)	5 µg/mL
Ultra-LEAF Purified anti-mouse IL-4 Antibody (clone 11B11)	Biolegend	(Cat# 504135, RRID:AB_ 2750404)	100 µg
Ultra-LEAF Purified anti-mouse IL-3 Antibody	Biolegend	(Cat# 503911, RRID: AB_2890852)	50 µg

Chemicals, peptides, and recombinant proteins		
Diphtheria Toxin, Unnicked, Corynebacterium diphtheriae - Calbiochem	Sigma-Aldrich	Catalog # 322326
DPBS, 10x, no calcium, no magnesium	Thermo Fisher Scientific	Catalog # 14200-067

PBS, pH 7.2	Thermo Fisher Scientific	Catalog # 20012-019
Imject Alum Adjuvant	Thermo Fisher Scientific	Catalog # 77161
Tween 20, 100% Nonionic Detergent	Bio-Rad	Catalog # 1706531; CAS: 9005-64-5
Sm/RNP Complex Antigen affinity purified	ImmunoVision	Catalog # SRC-3000
Deoxyribonucleic acid sodium salt from calf thymus	Sigma-Aldrich	Catalog # D1501; CAS: 73049-39-5
Goat Serum Donor Herd	Sigma-Aldrich	Catalog # G6767
Pierce DNA Coating Solution	Thermo Fisher Scientific	Catalog # 17250
Albumin from chicken egg white	Sigma-Aldrich	Catalog # A5503; CAS: 9006-59-1
Trypan Blue Solution, 0.4%	Thermo Fisher Scientific	Catalog # 15250-061
RPMI 1640 Medium, GlutaMAX Supplement, HEPES	Thermo Fisher Scientific	Catalog # 72400-021
MEM Non-Essential Amino Acids Solution (100X)	Thermo Fisher Scientific	Catalog # 11140-035
Fetal Bovine Serum, qualified, Brazil	Thermo Fisher Scientific	Catalog # 10270-106; Lot: 2275142
Penicillin - streptomycin (5 000 U/ml)	Thermo Fisher Scientific	Catalog # 15070-063
Sodium Pyruvate (100 mM)	Thermo Fisher Scientific	Catalog # 11360-039
2-Mercaptoethanol	Sigma-Aldrich	Catalog # M6250-100ML; CAS: 60-24-2
EDTA UltraPure 0.5M, pH de 8,0	Thermo Fisher Scientific	Catalog # 15575-020
Phorbol 12-myristate 13-acetate	Sigma-Aldrich	Catalog # P8139-1MG; CAS: 16561-29-8
Ionomycin from Streptomyces conglobatus	Sigma-Aldrich	Catalog # I9657-1MG; CAS: 56092-81-0
Brefeldin A	Sigma-Aldrich	Catalog # B7651; CAS: 20350-15-6
Pristane	Sigma-Aldrich	Catalog # P2870; CAS: 1921-70-6
Heparin sodium salt from porcine intestinal mucosa	Sigma-Aldrich	Catalog # H4784; CAS: 9041-08-1
Rat Sprague Dawley IgG Affinity Purified	Innovative Research	Catalog # IRTSDIGGAP50MG
Hamster Armenian IgG Affinity Purified	Innovative Research	Catalog # IHMARIGG50MG
Human IgG Affinity Purified	Innovative Research	Catalog # IHUIGGAP1000MG
Goat IgG Fractionated Purified Lyophilized	Innovative Research	Catalog # IGTIGGGFLY1GM
OCT embedding cryoembedding Matrix	Thermo Fisher Scientific	Catalog # LAMB/OCT
Ghost Dye Violet 510	Tonbo	Catalog # 13-0870-T100
Streptavidin, Alexa Fluor 594 conjugate	Thermo Fisher Scientific	Catalog # S11227
Streptavidin, Alexa Fluor 647 conjugate	Thermo Fisher Scientific	Catalog # S21374
Streptavidin, APC/Cy7 conjugate	Biolegend	Catalog # 405208
UltraComp eBeads Plus Compensation Beads	Thermo Fisher Scientific	Catalog # 01-3333-42
MagniSort Streptavidin Negative Selection Beads	Thermo Fisher Scientific	Catalog # MSNB-6002-74
Intracellular Staining Permeabilization Wash Buffer (10X)	Biolegend	Catalog # 421002
Fixation Buffer	Biolegend	Catalog # 420801
Recombinant Murine IL-3	Peprotech	Catalog # 213-13
Recombinant Human IL-3 (carrier-free)	Biolegend	Catalog # 578006

Recombinant Mouse PD-1 (CD279)-Fc Chimera (carrier-free)	Biolegend	Catalog # 764802
Recombinant Murine IL-4	Biolegend	Catalog # 574302
Prostaglandin D2	Cayman Chemicals	Catalog # 12010-366033
BD Cytofix/Cytoperm Kit	BD Biosciences	Cat# 554714
EasySep Mouse Pan-B Cell Isolation Kit	Stemcell Technologies	Cat# 19844
EasySep Mouse CD4+ T Cell Isolation Kit	Stemcell Technologies	Cat# 19852
Sulfo-NHS-LC-Biotine EZ-Link	Thermo Fisher Scientific	Cat# 21335
Commercial assays		
eBioscience Foxp3 / Transcription Factor Staining Buffer Set	Thermo Fisher Scientific	Catalog # 00-5523-00
EasySep Human Naïve CD4+ T Cell Isolation Kit	Stemcell Technologies	Catalog # 19555
EasySep Human Basophil Isolation Kit	Stemcell Technologies	Catalog # 17969
EasySep Mouse Naïve CD4+ T Cell Isolation Kit	Stemcell Technologies	Catalog # 19765
SsoAdvanced Universal SYBR Green Supermix	Bio-Rad	Catalog # 172-5274
iScript cDNA Synthesis Kit	Bio-Rad	Catalog # 170-8891
IL-3 Mouse ELISA Kit	Thermo Fisher Scientific	Catalog # EMIL3
Experimental models:		
Mouse: B6.129P2-Gt(ROSA)26$^{Sortm1(DTA)Lky/J}$	The Jackson Laboratory	RRID:IMSR_JAX:009669
Mouse: Il4$^{flox/flox}$ C57BL/6	Shibata et al. Proc Natl Acad Sci USA. 2018 Dec 18;115(51):13057-13062.	N/A
Mouse: Cd274loxP (PD-L1$^{flox/flox}$) C57BL/6	Schwartz et al. J Exp Med. 2017 Sep 4;214(9):2507-2521.	N/A
Mouse: Il-6$^{flox/flox}$ C57BL/6	Quintana et al. Brain Behav Immun. 2013 Jan;27(1):162-73.	N/A
Mouse: Mcpt8$^{Cre-tdTomato}$ C57BL/6	Tchen et al. Front Immunol. 2022 Jun 29;13:900532.	N/A
Mouse: Mcpt8^{DTR} C57BL/6	Wada et al. J Clin Invest. 2010 Aug;120(8):2867-75.	N/A
Mouse: B6.129S4-Lyn$^{tm1Sor/J}$	The Jackson Laboratory	RRID:IMSR_JAX:003515
Mouse: B6.129S4-Lyn$^{tm1Sor/J}$ Mcpt8^{DTR}	Pellefigues et al. Nat Commun. 2018 Feb 20;9(1):725.	N/A
Oligonucleotides		
Merck qPCR primers were KiCqStart SYBR Green Primers	Merck / Sigma-Aldrich	Cat. # KSPQ12012
Actin b Forward 5'- GATGTATGAAGGCTTTGGTC -3'	Merck / Sigma-Aldrich	Cat. # M_Actb_1
Actin b Reverse 5'- TGTGCACTTTTATTGGTCTC -3'	Merck / Sigma-Aldrich	Cat. # M_Actb_1
Batf Forward 5'- AAAATGACAAGTCAACCCTG -3'	Merck / Sigma-Aldrich	Cat. # M_Batf_1
Batf Reverse 5'- TTAGAAAACTATCCACCCCC -3'	Merck / Sigma-Aldrich	Cat. # M_Batf_1
Bcl6 Forward 5'- GAAACCTTACCATTGTGAGAAG -3'	Merck / Sigma-Aldrich	Cat. # M_Bcl6_1
Bcl6 Reverse 5'- GAACACTCCATGCTTCATTC -3'	Merck / Sigma-Aldrich	Cat. # M_Bcl6_1
Gata3 Forward 5'- TATTAACAGACCCCTGACTATG -3'	Merck / Sigma-Aldrich	Cat. # M_Gata3_1
Gata3 Reverse 5'- CACCTTTTTGCACTTTTTCG -3'	Merck / Sigma-Aldrich	Cat. # M_Gata3_1
Maf Forward 5'- CAAGGAGAAATACGAGAAGC -3'	Merck / Sigma-Aldrich	Cat. # M_Maf_1
Maf Reverse 5'- TCACATGAAAAATTCGGGAG -3'	Merck / Sigma-Aldrich	Cat. # M_Maf_1

Prdm1 Forward 5'- AACAGCAAAGAGGTTATTGG -3'	Merck / Sigma-Aldrich	Cat. # M_Prdm1_1
Prdm1 Reverse 5'- GGAACTCCTCTCTGGAATAG -3'	Merck / Sigma-Aldrich	Cat. # M_Prdm1_1
Cxcr5 Forward 5'-ATGACCTGTACAAGGAACTG-3'	Merck / Sigma-Aldrich	Cat. # M_Cxcr5_1
Cxcr5 Reverse 5'-CAGGATGTTTCCCATCATAC-3'	Merck / Sigma-Aldrich	Cat. # M_Cxcr5_1
Bach2 Forward 5'- TGTAGCCTTCTCATCTCTTCCT -3'	Integrated DNA Tech.	Cat. # Mm.PT.58.12332872
Bach2 Reverse 5'- ATCCACAGACATGCCGTTC -3'	Integrated DNA Tech.	
Software and algorithms		
FlowJo v10.8.1	Tree Star / BD Biosciences	FlowJo (RRID:SCR_00852)
BD FACSDiva Software v8.0	BD Biosciences	(RRID:SCR_00145)
Graphpad Prism v9.4 – v10.2	Graphpad	(RRID:SCR_00279)
ImageJ v1.43u and v1.54f	NIH - Schneider et al. Nat Methods. 2012 Jul;9(7):671-5.	(RRID:SCR_00307)
CellSens Dimensions 1.4 (Build 8583)	Olympus	(RRID:SCR_01455)
QuPath v.0.5	Queens University Belfast; Northern Ireland; UK – Bankhead et al. Sci Rep. 2017 Dec; 7(1): 16878.	(RRID:SCR_018257)
Zen 2.1 software (v11.0.4.19)	Zeiss	(RRID:SCR_013672)
Leica Application Suite X (v3.5.5.19976)	Leica	(RRID:SCR_013673)
Other		
BD LSRFortessa X-20 Cell Analyzer	BD Biosciences	N/A
BD FACSMelody Cell Sorter	BD Biosciences	(RRID:SCR_023209)
BD LSRFortessa Cell Analyzer	BD Biosciences	(RRID:SCR_019601)
Leica DM IRB Inverted microscope	Leica	N/A
Video camera microscope attachment	Hamamatsu	ORCA-03G02
CFX96 Touch Real-Time PCR Detection System	Bio-Rad	(RRID:SCR_018064)
Fluorescence microplate reader	TECAN	SPARK 10M
