# Supplementary material for: PD-L1- and IL-4-expressing basophils promote pathogenic accumulation of T follicular helper cells in lupus
Source: Nat Commun. 2024 Apr 22;15:3389. doi: 10.1038/s41467-024-47691-w (PMC11035650; doi:10.1038/s41467-024-47691-w)
Supplement: Supplementary file 3 — Reporting Summary [file 41467_2024_47691_MOESM3_ESM.pdf]

nature portfolio

Corresponding author(s): Nicolas CHARLES

Last updated by author(s): Mar 15, 2024

Reporting Summary

Nature Portfolio wishes to improve the reproducibility of the work that we publish. This form provides structure for consistency and transparency in reporting. For further information on Nature Portfolio policies, see our Editorial Policies and the Editorial Policy Checklist.

Statistics

For all statistical analyses, confirm that the following items are present in the figure legend, table legend, main text, or Methods section.

n/a	Confirmed	
☐	☒	The exact sample size (*n*) for each experimental group/condition, given as a discrete number and unit of measurement
☐	☒	A statement on whether measurements were taken from distinct samples or whether the same sample was measured repeatedly
☐	☒	The statistical test(s) used AND whether they are one- or two-sided<br>*Only common tests should be described solely by name; describe more complex techniques in the Methods section.*
☒	☐	A description of all covariates tested
☐	☒	A description of any assumptions or corrections, such as tests of normality and adjustment for multiple comparisons
☐	☒	A full description of the statistical parameters including central tendency (e.g. means) or other basic estimates (e.g. regression coefficient) AND variation (e.g. standard deviation) or associated estimates of uncertainty (e.g. confidence intervals)
☐	☒	For null hypothesis testing, the test statistic (e.g. *F*, *t*, *r*) with confidence intervals, effect sizes, degrees of freedom and *P* value noted<br>*Give P values as exact values whenever suitable.*
☒	☐	For Bayesian analysis, information on the choice of priors and Markov chain Monte Carlo settings
☒	☐	For hierarchical and complex designs, identification of the appropriate level for tests and full reporting of outcomes
☐	☒	Estimates of effect sizes (e.g. Cohen's *d*, Pearson's *r*), indicating how they were calculated

Our web collection on statistics for biologists contains articles on many of the points above.

Software and code

Policy information about availability of computer code

Data collection	For FACS data acquisition, BD FACSDiva Software v8.0 was used.<br>For immunofluorescence data acquisition, CellSens Dimensions 1.4 (Build 8583) (Olympus) was used. For confocal microscopy, images were acquired with a 4 lasers LSM 780 Zeiss confocal microscope with a x10 objective using Zen 2.1 software (v11.0.4.19) and with a 4 lasers Leica sp8 confocal microscope with a x63 objective using LAS X software (v3.5.5.19976).<br>For optical density measurements, Magellan software was used (TECAN, Spark 10M).<br>For qPCR data acquisition, Bio-Rad CFX96 Touch Real-Time PCR Detection System embedded software was used.
Data analysis	For flow cytometric analysis, FlowJo ver 10.8.1 (BD Biosciences) was used to analyze FACS data.<br>For statistical analysis, GraphPad Prism (ver 9.4 to 10.2) was used<br>For immunofluorescence quantification, ImageJ v1.43u (NIH) was used.<br>For qPCR analysis, Bio-Rad CFX manager software (ver 3.1) was used.<br>Confocal images processing was done using Image J software (v1.54f) and quantifications were done with QuPath software (v. 0.5)

For manuscripts utilizing custom algorithms or software that are central to the research but not yet described in published literature, software must be made available to editors and reviewers. We strongly encourage code deposition in a community repository (e.g. GitHub). See the Nature Portfolio guidelines for submitting code & software for further information.

Data

Policy information about availability of data

All manuscripts must include a data availability statement. This statement should provide the following information, where applicable:

- Accession codes, unique identifiers, or web links for publicly available datasets
- A description of any restrictions on data availability
- For clinical datasets or third party data, please ensure that the statement adheres to our policy

All data reported in this paper will be shared by the corresponding author upon reasonable request.

Research involving human participants, their data, or biological material

Policy information about studies with human participants or human data. See also policy information about sex, gender (identity/presentation), and sexual orientation and race, ethnicity and racism.

Reporting on sex and gender	204 patients with SLE participated to the study (SLE). Among them, 90% (n=187) were females.<br>43 healthy volunteers particpated to the study (HV). Among them, 74% (n=32) were females.
Reporting on race, ethnicity, or other socially relevant groupings	n/a
Population characteristics	Age, mean±SD, yr: SLE 38.87±12.4; HV 34±9.2.
Recruitment	Blood samples were collected from adult patients enrolled in a prospective long-term study of systemic lupus erythematosus (SLE) and chronic renal diseases. All SLE patients fulfilled the American College of Rheumatology (ACR) classification criteria for SLE. SLE and healthy control (CT) donor characteristics are shown in Supplementary Table S1.<br>inactive patients (SLEDAI 0/1) are mainly outpatients coming to the hospital for a usual care medical visit. The other patients are followed in the nephrology or in the Internal medicine department of the Bichat hospital and are seen by the physicians for the diagnosis of a flare (renal or not). Pregnant and/or HIV, HBV and HCV seropositive patients were excluded from the study to avoid any interference in the observed immunological phenotypes. The blood is drawn for analysis before any additional treatment is administered. For the nephrology department, after informed consent, blood is drawn and cells analyzed from all patients present at the hospital for a diagnostic kidney biopsy. The diagnostic is blinded to the operator and unblinded once the analysis are done and the cohort "frozen". For the samples from the internal medicine department, the diagnostic was known.
Ethics oversight	The study and the use of human material have been approved by the Comité Régional de Protection des Personnes (CRPP, Paris, France) under the reference ID-RCB 2014-A00809-38. SLE samples were obtained from in- and outpatients and clinical data were harvested after approval by the Commission Nationale de l'Informatique et des Libertés (CNIL). Healthy controls were recruited among volunteering healthcare workers and through the Etablissement Français du Sang (EFS). All samples were collected in heparinized tubes (BD vacutainer) and processed within 4 hours.<br>Written informed consent was obtained from all individuals.

Note that full information on the approval of the study protocol must also be provided in the manuscript.

Field-specific reporting

Please select the one below that is the best fit for your research. If you are not sure, read the appropriate sections before making your selection.

☒ Life sciences ☐ Behavioural & social sciences ☐ Ecological, evolutionary & environmental sciences

For a reference copy of the document with all sections, see nature.com/documents/nr-reporting-summary-flat.pdf

Life sciences study design

All studies must disclose on these points even when the disclosure is negative.

Sample size	No statistical methods were used to determine sample size.<br>Concerning Human data, the acquired database was frozen and analyzed as presented in the figures.<br>For cTFH analysis, a number of patients > 10 per group (inactive, mild and active) was deemed necessary to account for the disease heterogeneity. The sample size is similar to other research in the field.<br>For basophil analysis, the patients analyzed were all the individuals enrolled in the study from 04/2015 to 02/2020.<br>Concerning mouse experiments, the sample size was more than 3 per group. Based on the 3R principle, the minimal replicate number sufficient to ascertain statistics by unpaired t-test, one-way ANOVA, or two-way ANOVA was used.<br>For human and murine co-culture experiments, basophils and T cells from individual donors were combined to optimize the biological replicate numbers.
Data exclusions	No data were excluded from experiments.

Replication	All the experiments were successfully replicated at least two times completely independently with at least 2 samples per experimental and control groups. Most of the experiments were repeated more than 3 times as indicated in figure legends.
Randomization	For ex vivo and in vivo experiments, purified cells (either human or murine) and animals used in this study were randomly assigned to their respective groups before the experiments were performed. For basophil depletion or pristane-induced lupus-like models, DT, PBS and/or pristane-injected mice were kept in the same cage and randomly attributed to their respective groups.
Blinding	Blinding was not achieved in mouse experiments due to animal identification and labeling for treatment purposes. However, acquired data were analyzed in a blinded way before being unblinded to attribute data to the corresponding experimental group.

Reporting for specific materials, systems and methods

We require information from authors about some types of materials, experimental systems and methods used in many studies. Here, indicate whether each material, system or method listed is relevant to your study. If you are not sure if a list item applies to your research, read the appropriate section before selecting a response.

Materials & experimental systems

n/a	Involved in the study
☐	☒ Antibodies
☒	☐ Eukaryotic cell lines
☒	☐ Palaeontology and archaeology
☐	☒ Animals and other organisms
☒	☐ Clinical data
☒	☐ Dual use research of concern
☒	☐ Plants

Methods

n/a	Involved in the study
☒	☐ ChIP-seq
☐	☒ Flow cytometry
☒	☐ MRI-based neuroimaging

Antibodies

Antibodies used

All antibodies were used following manufacturers' instructions and titrations to determine the best dilution to use for each lot was determined in real conditions before use in experimental settings.
Alexa Fluor 488 anti-mouse Complement Component C3 (Clone: 11H9) Santa Cruz Biotechnology (Cat# sc-58926, RRID:AB_1119819)
Alexa Fluor 488 anti-mouse/human CD44 (Clone: IM7) Biolegend (Cat# 103016, RRID:AB_493679)
Alexa Fluor 488 anti-mouse IgG Fcγ fragment specific (Goat Polyclonal) Jackson ImmunoResearch (Cat# 115-545-008, RRID:AB_2338842)
Alexa Fluor 488 anti-mouse CD25 (Clone: PC61) Biolegend (Cat# 102017, RRID:AB_493333)
Alexa Fluor 647 anti-mouse FcεRIα (Clone: MAR-1) Biolegend (Cat# 134310, RRID:AB_1626093)
Alexa Fluor 647 anti-mouse CD3ε (Clone: 145-2C11) Biolegend (Cat# 100322, RRID:AB_389322)
Alexa Fluor 647 anti-mouse IgG (Clone: Poly4053) Biolegend (Cat#405322, RRID:AB_2563045)
Alexa Fluor 700 anti-mouse CD45 (Clone: 30-F11) Biolegend (Cat# 103128, RRID:AB_493715)
Alexa Fluor 700 anti-mouse CD62L (Clone: MEL-14) Biolegend (Cat# 104426, RRID:AB_493719)
APC/Fire 750 anti-mouse CD4 (Clone: RM4-5) Biolegend (Cat# 100568, RRID:AB_2629699)
APC/Cyanine7 anti-mouse CD4 (Clone: RM4-5) Biolegend (Cat# 100526, RRID:AB_312727)
APC/Cyanine7 anti-mouse TCR β chain (Clone: H57-597) Biolegend (Cat# 109220, RRID:AB_893624)
APC/Fire 750 anti-mouse TCR β chain (Clone: H57-597) Biolegend (Cat# 109246, RRID:AB_2629697)
APC/Cyanine7 anti-mouse CD19 (Clone: 6D5) Biolegend (Cat# 115530, RRID:AB_830707)
APC/Fire 750 anti-mouse CD19 (Clone: 6D5) Biolegend (Cat# 115558, RRID:AB_2572120)
APC/Cyanine7 anti-mouse CD117 (c-kit) (Clone: 2B8) Biolegend (Cat# 105826, RRID:AB_1626278)
APC/Fire 750 anti-mouse CD117 (c-Kit) (Clone: 2B8) Biolegend (Cat# 105838, RRID:AB_2616739)
Biotin anti-mouse CD185 (CXCR5) (Clone: L138D7) Biolegend (Cat# 145510, RRID:AB_2562126)
Biotin anti-mouse CD4 (Clone: RM4-5) Biolegend (Cat# 100508, RRID:AB_312711)
Biotin anti-mouse CD19 (Clone: 6D5) Biolegend (Cat# 115504, RRID:AB_313639)
Biotin anti-mouse CD8a (Clone: 53-6.7) Biolegend (Cat# 100704, RRID:AB_312743)

Biotin anti-mouse NK-1.1 (Clone: PK136) Biolegend (Cat# 108704, RRID:AB_313391)
BUV395 anti-mouse CD45 (Clone: 30-F11) BD Biosciences (Cat# 564279, RRID:AB_2651134)
Brilliant Violet 421 anti-mouse CD4 (Clone: GK1.5) Biolegend (Cat# 100438, RRID:AB_11203718)
Brilliant Violet 421 anti-mouse CD279 (PD-1) (Clone: 29F.1A12) Biolegend (Cat# 135218, RRID:AB_2561447)
Brilliant Violet 421 anti-mouse CD138 (Syndecan-1) (Clone: 281-2) Biolegend (Cat# 142508, RRID:AB_11203544)
Brilliant Violet 421 anti-mouse/human CD44 (Clone: IM7) Biolegend (Cat# 103040, RRID:AB_2616903)
Brilliant Violet 605 anti-mouse CD45 (Clone: 30-F11) Biolegend (Cat# 103140, RRID:AB_2562342)
Brilliant Violet 605 anti-mouse CD279 (PD-1) (Clone: 29F.1A12) Biolegend (Cat# 135220, RRID:AB_2562616)
Brilliant Violet 605 anti-mouse CD152 (Clone: UC10-4B9) Biolegend (Cat# 106323, RRID:AB_2566467)
Brilliant Violet 785 anti-mouse CD19 (Clone: 6D5) Biolegend (Cat# 115543, RRID:AB_11218994)

Brilliant Violet 785 anti-mouse/human CD44 (Clone: IM7) Biolegend (Cat# 103059, RRID:AB_2571953)
Brilliant Violet 785 anti-mouse CD274 (B7-H1, PD-L1) (Clone: 10F.9G2) Biolegend (Cat# 124331, RRID:AB_2629659)
eFluor 450 anti-mouse IL-6 (Clone: MP5-20F3) Thermo Fisher Scientific (Cat# 48-7061-82, RRID:AB_2574103)
eFluor 450 anti-mouse IL-21 (Clone: FFA21) Thermo Fisher Scientific (Cat# 48-7211-82, RRID:AB_2811832)
FITC anti-mouse Complement Component C3 (Clone: RmC11H9) Cedarlane (Cat# CL7503F, RRID:AB_10061294)
FITC anti-mouse IgM (Goat polyclonal) BioRad (AbD Serotec) (Cat# 102002, RRID:AB_619870)
FITC anti-mouse CD123 (Clone: 5B11) Thermo Fisher Scientific (Cat# 11-1231-82, RRID:AB_465192)
FITC anti-mouse CD49b (Clone: HMα2) Biolegend (Cat# 103504, RRID:AB_313027)
FITC anti-mouse IL-6 (Clone: MP5-20F3) Thermo Fisher Scientific (Cat# 11-7061-82, RRID:AB_465394)
FITC anti-mouse TNF-α (Clone: MP6-XT22) R&D Systems (Cat# IC410F, RRID:AB_357323)
FITC anti-mouse IgA (Polyclonal IgG) BioRad (AbD Serotec) (Cat#STAR137, RRID:AB_2075638)
Pacific Blue anti-mouse CD49b (pan-NK cells) (Clone: DX5) Biolegend (Cat# 108918, RRID:AB_2265144)
PE anti-mouse IL-21 (Clone: FFA21) Thermo Fisher Scientific (Cat# 12-7211-82, RRID:AB_1834466)
PE anti-mouse CD278 (ICOS) (Clone: 15F9) Biolegend (Cat# 107706, RRID:AB_313335)
PE anti-mouse FOXP3 (Clone: FJK-16s) Thermo Fisher Scientific (Cat# 12-5773-82, RRID:AB_465936)
PE-CF594 anti-mouse IL-4 (Clone: 11B11) BD Biosciences (Cat# 562450, RRID:AB_2737616)
PE/Cyanine7 anti-mouse CD45 (Clone: 30-F11) Biolegend (Cat# 103114, RRID:AB_312979)
PE/Cyanine7 anti-mouse IL-13 (Clone: eBio13A) Thermo Fisher Scientific (Cat# 25-7133-82, RRID:AB_2573530)
PE/Cyanine7 anti-mouse CD62L (Clone: MEL-14) Biolegend (Cat# 104418, RRID:AB_313103)
PE/Cyanine7 anti-mouse CD274 (B7-H1, PD-L1) (Clone: 10F.9G2) Biolegend (Cat# 124314, RRID:AB_10643573)
PE/Dazzle 594 anti-mouse CD279 (PD-1) (Clone: 29F.1A12) Biolegend (Cat# 135228, RRID:AB_2566006)
PE/Dazzle 594 anti-mouse CD19 (Clone: 6D5) Biolegend (Cat# 115554, RRID:AB_2564001)
PE/Dazzle 594 anti-mouse/human CD44 (Clone: IM7) Biolegend (Cat#103056, RRID:AB_2564044)
PerCP/Cyanine5.5 anti-mouse CD8a (Clone: 53-6.7) Biolegend (Cat# 100734, RRID:AB_2075238)
PerCP/Cyanine5.5 anti-mouse CD4 (Clone: RM4-5) Biolegend (Cat# 100540, RRID:AB_893326)
PerCP/Cyanine5.5 anti-mouse IFN-γ (Clone: XMG1.2) Biolegend (Cat# 505822, RRID:AB_961359)
PerCP/eFluor710 anti-mouse IgM (Clone: II/41) Thermo Fisher Scientific (Cat#46-5790-82, RRID:AB_1834435)
PerCP/eFluor710 anti-mouse CD200R (Clone: OX110) Thermo Fisher Scientific (Cat#46-5201-82, RRID: AB_10804765)
Alexa Fluor 647 anti-human CD294 (CRTH2) (Clone: BM16) Biolegend (Cat# 350104, RRID:AB_10642025)
Alexa Fluor 647 anti-human CD273 (B7-DC, PD-L2) (Clone: MIH18) Biolegend (Cat# 345514, RRID:AB_2728313)
Alexa Fluor 700 anti-human/mouse/rat CD278 (ICOS) (Clone: C398.4A) Biolegend (Cat# 313528, RRID:AB_2566126)
APC anti-human CD4 (Clone: RPA-T4) Biolegend (Cat# 300537, RRID:AB_2562051)
APC anti-human CD275 (B7-H2, ICOSL) (Clone: 2D3) Biolegend (Cat# 309408, RRID:AB_2565557)
APC/Cyanine7 anti-human CD183 (CXCR3) (Clone: G025H7) Biolegend (Cat# 353722, RRID:AB_2561423)
Brilliant Violet 421 anti-human CD203c (E-NPP3) (Clone: NP4D6) Biolegend (Cat# 324612, RRID:AB_2563848)
Brilliant Violet 421 anti-human CD274 (B7-H1, PD-L1) (Clone: 29E.2A3) Biolegend (Cat# 329714, RRID:AB_2563852)
Brilliant Violet 421 anti-human CD279 (PD-1) (Clone: NAT105) Biolegend (Cat# 367422, RRID:AB_2721517)
Brilliant Violet 605 anti-human CD193 (CCR3) (Clone: 5E8) Biolegend (Cat# 310716, RRID:AB_2563831)
Brilliant Violet 785 anti-human CD197 (CCR7) (Clone: G043H7) Biolegend (Cat# 353230, RRID:AB_2563630)
BUV395 anti-human CD3 (Clone: SK7 (also known as Leu-4)) BD Biosciences (Cat# 564000, RRID:AB_2744382)
BUV395 anti-human CD14 (Clone: MϕP9 (also known as MϕP-9)) BD Biosciences (Cat# 563561, RRID:AB_2744288)
BUV395 anti-human CD56 (Clone: NCAM16.2 (also known as NCAM 16)) BD Biosciences (Cat# 563554, RRID:AB_2687886)
BUV395 anti-human CD19 (Clone: SJ25C1 (also known as SJ25-C1)) BD Biosciences (Cat# 563551, RRID:AB_2738274)
PE anti-human CD185 (CXCR5) (Clone: J252D4) Biolegend (Cat# 356904, RRID:AB_2561813)
PE anti-human CD84 (Clone: CD84.1.21) Biolegend (Cat# 326008, RRID:AB_2229003)
PE anti-human CD252 (OX40L) (Clone: 11C3.1) Biolegend (Cat# 326308, RRID:AB_2207271)
PE/Cyanine7 anti-human FcεRIα (Clone: AER-37 (CRA-1)) Biolegend (Cat# 334620, RRID:AB_10575314)
PE/Dazzle 594 anti-human CD123 (Clone: 6H6) Biolegend (Cat# 306034, RRID:AB_2566450)
Alexa Fluor 488 Rat IgG2a, κ Isotype Control (Clone: RTK2758) Biolegend (Cat# 400525, RRID:AB_2864283)
Alexa Fluor 488 Goat IgG whole molecule (Goat polyclonal) Jackson ImmunoResearch (Cat# 005-540-003, RRID:AB_2337013)
Alexa Fluor 488 Rat IgG2b, κ Isotype Ctrl (Clone: RTK4530) Biolegend (Cat# 400625, RRID:AB_389321)
Alexa Fluor 647 Armenian Hamster IgG Isotype Control (Clone: HTK888) Biolegend (Cat# 400924, RRID:AB_2922967)
Alexa Fluor 647 Rat IgG2a, κ Isotype Control (Clone: RTK2758) Biolegend (Cat# 400526, RRID:AB_2864284)
Alexa Fluor 647 Mouse IgG1, κ Isotype Control (Clone: MOPC-21) Biolegend (Cat# 400130, RRID:AB_2800436)
Alexa Fluor 700 Rat IgG2a, κ Isotype Control (Clone:
RTK2758) Biolegend (Cat# 400528, RRID:AB_2923249)
Alexa Fluor 700 Rat IgG2b, κ Isotype Control (Clone: RTK4530) Biolegend (Cat# 400628, RRID:AB_493783)
Alexa Fluor 700 Mouse IgG1, κ Isotype Control (Clone: MOPC-21) Biolegend (Cat# 400143, RRID:AB_2923250)
APC Mouse IgG1, κ Isotype Control (Clone: MOPC-21) Biolegend (Cat# 400119, RRID:AB_2888687)
APC Mouse IgG2b, κ Isotype Control (Clone: MPC-11) Biolegend (Cat# 400322, RRID:AB_326500)
APC/Cyanine7 Armenian Hamster IgG Isotype Control (Clone: HTK888) Biolegend (Cat# 400927, RRID:AB_2923251)
APC/Cyanine7 Rat IgG2a, κ Isotype Control (Clone: RTK2758) Biolegend (Cat# 400523, RRID:AB_2923252)
APC/Cyanine7 Rat IgG2b, κ Isotype Control (Clone: RTK4530) Biolegend (Cat# 400623, RRID:AB_326565)
APC/Cyanine7 Mouse IgG1, κ Isotype Control (Clone: MOPC-21) Biolegend (Cat# 400127, RRID:AB_2892538)
APC/Fire 750 Armenian Hamster IgG Isotype Control (Clone: HTK888) Biolegend (Cat# 400961, RRID:AB_2923253)
APC/Fire 750 Rat IgG2a, κ Isotype Control (Clone: RTK2758) Biolegend (Cat# 400567, RRID:AB_2923254)
APC/Fire 750 Rat IgG2b, κ Isotype Control (Clone: RTK4530) Biolegend (Cat# 400669, RRID:AB_2905475)
Biotin Rat IgG2b, κ Isotype Control (Clone: RTK4530) Biolegend (Cat# 400603, RRID:AB_326547)

BUV395 Rat IgG2b, κ Isotype Control (Clone: R35-38) BD Biosciences (Cat# 563560, RRID:AB_2869507)
BUV395 Mouse IgG1, k Isotype Control (Clone: X-40) BD Biosciences (Cat# 563547, RRID:AB_2869503)
BUV395 Mouse IgG2b, κ Isotype Control (Clone: 27-35) BD Biosciences (Cat# 563558, RRID:AB_2869505)
Brilliant Violet 421 Rat IgG2a, κ Isotype Control (Clone: RTK2758) Biolegend (Cat# 400535, RRID:AB_10933427)
Brilliant Violet 421™ Rat IgG2b, κ Isotype Control (Clone: RTK4530) Biolegend (Cat# 400639, RRID:AB_10895758)
Brilliant Violet 421 Mouse IgG1, κ Isotype Control (Clone: MOPC-21) Biolegend (Cat# 400157, RRID:AB_10897939)
Brilliant Violet 421 Mouse IgG2b, κ Isotype Control (Clone: MPC-11) Biolegend (Cat# 400341, RRID:AB_10898160)
Brilliant Violet 605 Armenian Hamster IgG Isotype Control (Clone: HTK888) Biolegend (Cat# 400943, RRID:AB_2923255)
Brilliant Violet 605 Rat IgG2a, κ Isotype Control (Clone: RTK2758) Biolegend (Cat# 400539, RRID:AB_11126979)
Brilliant Violet 605 Rat IgG2b, κ Isotype Control (Clone : RTK4530) Biolegend (Cat# 400649, RRID:AB_2864282)
Brilliant Violet 785 Rat IgG2a, κ Isotype Control (Clone: RTK2758) Biolegend (Cat# 400545, RRID:AB_11218993)
Brilliant Violet 785 Rat IgG2b, κ Isotype Control (Clone: RTK4530) Biolegend (Cat# 400647, RRID:AB_2923256)
Brilliant Violet 785 Mouse IgG2a, κ Isotype Control (Clone: MOPC-173) Biolegend (Cat# 400273, RRID:AB_2923257)
eFluor 450 Rat IgG1, κ Isotype Control (Clone: eBRG1) Thermo Fisher Scientific (Cat# 48-4301-82, RRID:AB_1271984)
eFluor 450 Rat IgG2a, κ Isotype Control (Clone: eBRG1) Thermo Fisher Scientific (Cat# 48-4321-82, RRID:AB_1271999)
FITC Armenian Hamster IgG Isotype Control (Clone: HTK888 Biolegend (Cat# 400905, RRID:AB_2923258)
FITC Rat IgG1, κ Isotype Control (Clone: RTK2071) Biolegend (Cat# 400405, RRID:AB_326511)
FITC Rat IgG2a, κ Isotype Control (Clone: RTK2758) Biolegend (Cat# 400506, RRID:AB_2736919)
Pacific Blue Rat IgM, κ Isotype Control (Clone: RTK2118) Biolegend (Cat# 400816, RRID:AB_10644001)
PE Mouse IgG1, κ Isotype Control (Clone: MOPC-21) Biolegend (Cat# 400112, RRID:AB_2847829)
PE Mouse IgG2a, κ Isotype Ctrl Antibody (Clone: MOPC-173) Biolegend (Cat# 400212, RRID:AB_326460)
PE-CF594 Rat IgG1, κ Isotype Control (Clone: R3-34) BD Biosciences (Cat# 562309, RRID:AB_11153318)
PE/Cyanine7 Rat IgG1, κ Isotype Control (Clone:
RTK2071) Biolegend (Cat# 400416, RRID:AB_326522)
PE/Cyanine7 Rat IgG2a, κ Isotype Control (Clone: RTK275) Biolegend (Cat# 400522, RRID:AB_326542)
PE/Cyanine7 Rat IgG2b, κ Isotype Control (Clone: RTK4530) Biolegend (Cat# 400618, RRID:AB_326560)
PE/Cyanine7 Mouse IgG2b, κ Isotype Control (Clone: MPC-11) Biolegend (Cat# 400325, RRID:AB_2923259)
PE/Dazzle 594 Rat IgG2a, κ Isotype Control (Clone: RTK2758) Biolegend (Cat# 400557, RRID:AB_2923260)
PE/Dazzle 594 Mouse IgG1, κ Isotype Control (Clone: MOPC-21) Biolegend (Cat# 400175, RRID:AB_2923261)
PerCP/Cyanine5.5 Rat IgG2a, κ Isotype Control (Clone: RTK2758) Biolegend (Cat# 400531, RRID:AB_2864286)
PerCP/Cyanine5.5 Rat IgG1, κ Isotype Control (Clone: RTK2071) Biolegend (Cat# 400425, RRID:AB_893689)
Anti-Mouse IgG (H+L) Cross-Adsorbed Secondary Antibody HRP coupled (Polyclonal) Thermo Fisher Scientific (Cat# G-21040, RRID:AB_2536527)
Anti-Mouse IgM Heavy Chain Antibody HRP Conjugated (Polyclonal) Bethyl Laboratories Cat# A90-101P, RRID:AB_67189)
Anti-mouse CD16/CD32 (Clone: 2.4G2) BioXCell (Cat# BE0307, RRID:AB_2736987)
Anti-mouse CD3ε F(ab')2 fragment (Clone: 145-2C11) BioXCell (Cat# BE0001-1FAB, RRID:AB_2687679)
Anti-mouse CD28 (Clone: PV-1) BioXCell (Cat# BE0015-5, RRID:AB_1107628)
Anti-human CD3 (Clone: OKT3) Thermo Fisher Scientific (Cat# 16-0037-81, RRID:AB_468854)Purified Anti-mouse CD279 (PD-1) (Clone: 29F.1A12) Biolegend (Cat# 135202, RRID:AB_1877121)
Anti-human/monkey CD28 (Clone: CD28.2) BioXCell (Cat# BE0291, RRID:AB_2687814)
Purified Anti-human CD279 (PD-1) (Clone: EH12.2H7) Biolegend (Cat# 329902, RRID:AB_940488)
Purified Mouse IgG1, κ Isotype Control (Clone: MOPC-21) Biolegend (Cat# 400102, RRID:AB_2891079)
Purified Anti-human IL-6 (Clone: MQ2-39C3) Biolegend (Cat# 501204, RRID:AB_2296206)
Purified Anti-human IL-4 Antibody (Clone: MP4-25D2) Biolegend (Cat# 500802, RRID:AB_315121)
Purified Rat IgG1, κ Isotype Control (Clone: RTK2071) Biolegend (Cat# 400402, RRID:AB_326508)
Purified anti-mMCP-8 Antibody Biolegend (Cat# 647402, RRID: AB_2290790)
Ultra-LEAF Purified anti-mouse IL-4 Antibody (clone 11B11) Biolegend (Cat# 504135, RRID:AB_ 2750404)
Ultra-LEAF Purified anti-mouse IL-3 Antibody Biolegend (Cat# 503911, RRID: AB_2890852)

Validation

All validation data are available on the manufacturers'websites under the corresponding catalog number.
All antibodies used in flow cytometry and immunofluorescence experiments were validated on the species studied for use in cytometry by the manufacturer.
https://www.biolegend.com/
https://www.bdbiosciences.com/
https://www.bio-rad-antibodies.com/
https://www.thermofisher.com/antibody/primary/query/*

Animals and other research organisms

Policy information about studies involving animals; ARRIVE guidelines recommended for reporting animal research, and Sex and Gender in Research

Laboratory animals

For spontaneous lupus-like model Lyn-/-, mice were analyzed between 30 and 40 weeks of age.
For pristane-induced lupus-like disease, mice were injected between 7 and 10 weeks of age and analyzed either 8 or 24 weeks after pristane injection as indicated in figure legends. Mice in immunization experiments were between 10 and 15 weeks of age.
Mcpt8DTR, Lyn-/- (B6.129S4-Lyntm1Sor/J, Stock# 003515)x Mcpt8DTR, Il4fl/fl , Il6fl/fl and Pdl1fl/fl mice were on a pure C57BL/6J

	background and bred in our animal facilities. RB6.129P2-Gt(ROSA)26Sortm1(DTA)Lky/J mice were purchased from The Jackson Laboratory through Charles River Laboratories (Stock# 009669). CT-M8 (Mcpt8tm1.1(cre)lcs or Mcpt8CT/CT or Mcpt8CT/+) mice were recently described. The mice crossed in our animal facilities (Mcpt8CT/+; Mcpt8CT/+ Il4fl/fl; Mcpt8CT/+ Il6fl/fl; Mcpt8CT/+ Pdl1fl/fl and Mcpt8CT/+ R26 DTA/+ were on a C57BL6J/N mixed genetic background at the F2 generation. Mice were maintained under specific pathogen-free conditions in our animal facilities with access to tap water and chow ad libidum, 12 hours dark/light cycles, an ambient temperature kept between 20°C and 24°C and a 55±10 % hygrometry. All mice were euthanized by CO2 inhalation in a regulated chamber (TemSega, France).
Wild animals	The study did not involved wild animals.
Reporting on sex	For lupus-like disease analysis of the Lyn—/— model, “aged” Mcpt8DTR and Lyn—/— Mcpt8DTR age-matched and sex-matched mice were analyzed between 30 and 45 weeks of age (50% males and 50 % females).<br>Pristane-induced lupus-like disease was initiated by injecting 500 µL of 2,6,10,14-tetramethyl-pentadecane or Pristane (Sigma) into the peritoneal cavity of 7-10 weeks-old female mice. Mice were analyzed 8 or 24 weeks after injection.
Field-collected samples	The study did not involve samples collected from the field.
Ethics oversight	The study was conducted in accordance with the French and European guidelines and approved by the local ethics committee comité d'éthique Paris Nord N°121 and the Ministère de l’enseignement supérieur, de la recherche et de l’innovation under the authorization number APAFIS#14115.

Note that full information on the approval of the study protocol must also be provided in the manuscript.

Flow Cytometry

Plots

Confirm that:

- [x] The axis labels state the marker and fluorochrome used (e.g. CD4-FITC).
- [x] The axis scales are clearly visible. Include numbers along axes only for bottom left plot of group (a 'group' is an analysis of identical markers).
- [x] All plots are contour plots with outliers or pseudocolor plots.
- [x] A numerical value for number of cells or percentage (with statistics) is provided.

Methodology

Sample preparation	Human samples handling<br>Heparinized human blood samples were centrifuged at 600 g for 5 minutes and 2 mL of plasma were collected and stored at -80°C for later analysis. Red blood cells (RBC) were lysed in RBC lysing buffer (150 mM NH4Cl, 12 mM NaHCO3, 1 mM EDTA, pH 7.4) in a ratio of 5mL of blood for 20mL of ACK lysing buffer. After 5 min of incubation at room temperature (rt), 25mL of PBS were added and cells were centrifuged at 600 g for 5 min, and the supernatant was discarded. This procedure was repeated 3 times. Leukocytes were then resuspended in fluorescence-activated cell sorting (FACS) buffer (PBS 1% BSA, 0.01% NaN3, 1mM EDTA) and prepared for flow cytometry (see below). Leukocyte count and viability (>95%) were assessed by trypan-blue staining on a Malassez hemacytometer.<br><br>Mouse samples processing<br>Mice were euthanized in a controlled CO2 chamber (TEM Sega) and blood sampling was performed through cardiac puncture with a heparin-coated syringe with a 25G needle. Blood was centrifuged at 300 g for 15 min and plasma was harvested and kept at -80°C for later analysis. RBC were lysed in 5mL of RBC lysing buffer for 5 min at rt and washed with 10mL of PBS. This procedure was repeated 3 times and cells were resuspended in PBS. The left kidney was harvested and embedded in OCT embedding matrix (Cellpath) and snap-frozen in liquid nitrogen and kept at -80°C for later analysis. Spleen and lymph nodes (cervical, brachial and inguinal) were harvested in PBS and dissociated by mechanical disruption on a 40 µm cell strainer (Falcon, Corning). For splenocytes, RBC were lysed once in 5mL RBC lysing buffer 5 min at rt and washed with 10mL of PBS. Cell counts were assessed by trypan-blue staining on a Malassez hemacytometer and 1 to 5 million cells were used per FACS staining condition.<br>Ex vivo stimulation of splenocytes.<br>Mouse splenocytes were harvested as described above and resuspended at 5 million cells/mL in culture medium (RPMI 1640 with Glutamax and 20mM HEPES, 1mM Na-pyruvate, non-essential amino acids 1X (all from Life Technologies), 100 µg/mL streptomycin and 100 U/mL penicillin (GE Healthcare) and 37.5 µM β-mercaptoethanol (Sigma-Aldrich) supplemented with 20% heat-inactivated fetal calf serum (FCS) (Life Technologies)). For phorbol-myristate-acetate (PMA) and ionomycin stimulation experiments, whole splenocytes were stimulated or not with 40 nM of PMA and 800 nM ionomycin for 4 hours in the presence of 2 µg/mL of brefeldin A (all from Sigma Aldrich, Merck) and cultured at 37°C and 5% CO2. For IL-3, IL-4 or anti-IgE stimulations, cells were stimulated with the doses indicated in the figure legends for 2 or 20 hours at 37°C and 5% CO2. Then, cells were harvested by repeated flushing, and wells were washed with 1mL of PBS. Samples were then prepared for flow cytometry analysis.<br>Flow cytometry staining<br>For human leukocytes, non-specific antibody binding sites were saturated with 20 µL of a solution containing 100 µg/mL of human, mouse, rat, and goat IgG (Jackson ImmunoResearch Europe and Innovative Research Inc.) in FACS buffer. 200 µL of staining solution containing the panel of fluorophore-conjugated specific antibodies or their fluorophore-conjugated isotypes (described in table S3) were added to the cells for 30 min at 4°C protected from light. After a wash in PBS, cells were fixed in fixation buffer (Biolegend) for 20 minutes at 4°C and then washed in FACS buffer before data acquisition. For mouse samples, cells washed in PBS were stained with GHOST 510 viability dye (TONBO) following the manufacturer’s instructions. Non-

	specific antibody binding sites were saturated with 10 µg/mL of anti-CD16/CD32 antibody clone 2.4G2 (BioXCell), and 100 µg/mL of polyclonal rat, mouse, and Armenian Hamster IgG (Innovative Research Inc.) in FACS buffer and stained with the antibodies described in Supplementary Table 3 for 30 min in the dark at 4°C. Cells were then washed in FACS Buffer before data acquisition. For intracellular staining, cells were first stained extracellularly as described above. Cells were washed in PBS and fixed with fixation buffer for 20 min at 4°C. Cell permeabilization and intracellular staining were realized with permeabilization/wash buffer (Biolegend) following the manufacturer's instructions. Cells were then resuspended in FACS buffer before acquisition
Instrument	All flow cytometry acquisitions were realized using a Becton Dickinson 5 lasers LSR Fortessa X-20.
Software	Collection: BD FACSDiva Software v8.0 (BD Biosciences)<br>Analysis: FlowJo ver 10.8.1 (BD Biosciences)
Cell population abundance	All purified cell populations (basophils and naive CD4 T cells from both human and mice) were tested for purity. All cells used were at least 93% pure.
Gating strategy	For all FACS acquisitions, singlets were selected on FSC-H/FSC-A and SSC-W/SSC-A plots.<br>Gating strategy for Human cells:<br>cTFH cells: CD3+ CD4+ CXCR5+ ICOS+ PD-1+ cells; cTFH1: CCR6– CXCR3+ cTFH cells; cTFH2: CCR6– CXCR3– cTFH cells; cTFH17: CCR6+ CXCR3– cTFH cells.<br>Basophils: SSClo CD14– CD3– CD19– CD56– CD123+ FcεRIα+ CCR3+ CRTH2+ cells.<br><br>Gating strategy for mouse cells:<br>Basophils: CD45lo TCRβ– CD19– CD117– CD49b+ CD123+ FcεRIα+ cells or CD45lo TCRβ– CD19– CD117– CD49b+ Tdt+ cells<br>TFH cells : CD45+ CD19– TCRβ+ CD8α– CD4+ CD44+ PD-1+ CXCR5+ cells<br>TH cells (non TFH): CD45+ CD19– TCRβ+ CD8α– CD4+ CD44+ CXCR5– cells<br>Plasmablasts: CD45+ TCRβ– CD19+ CD138+<br>GC B cells: CD45+ TCRβ– CD19+ CD138– IA-IEhi GL7hi (CD95+)<br>non B cell antigen presenting cells: CD45+TCRβ– CD19– IA-IEhi CD11c+

☒ Tick this box to confirm that a figure exemplifying the gating strategy is provided in the Supplementary Information.
